# Embryonic alcohol exposure disrupts the ubiquitin-proteasome system

**DOI:** 10.1172/jci.insight.156914

**Published:** 2022-12-08

**Authors:** Olivia Weeks, Bess M. Miller, Brian J. Pepe-Mooney, Isaac M. Oderberg, Scott H. Freeburg, Colton J. Smith, Trista E. North, Wolfram Goessling

**Affiliations:** 1 Division of Genetics, Brigham and Women’s Hospital, Harvard Medical School, Boston, Massachusetts, USA.; 2 Stem Cell Program, Department of Hematology/Oncology, Boston Children’s Hospital, Harvard Medical School, Boston, Massachusetts, USA.; 3 Harvard Stem Cell Institute, Cambridge, Massachusetts, USA.; 4 Broad Institute of MIT and Harvard, Cambridge, Massachusetts, USA.; 5 Harvard-MIT Division of Health Sciences and Technology, Cambridge, Massachusetts, USA.; 6 Division of Gastroenterology, Massachusetts General Hospital, Boston, Massachusetts, USA.

**Keywords:** Development, Cell stress, Embryonic development, Molecular biology

## Abstract

Ethanol (EtOH) is a commonly encountered teratogen that can disrupt organ development and lead to fetal alcohol spectrum disorders (FASDs); many mechanisms of developmental toxicity are unknown. Here, we used transcriptomic analysis in an established zebrafish model of embryonic alcohol exposure (EAE) to identify the ubiquitin-proteasome system (UPS) as a critical target of EtOH during development. Surprisingly, EAE alters 20S, 19S, and 11S proteasome gene expression and increases ubiquitylated protein load. EtOH and its metabolite acetaldehyde decrease proteasomal peptidase activity in a cell type–specific manner. Proteasome 20S subunit β 1 (*psmb1^hi2939Tg^*) and proteasome 26S subunit, ATPase 6 (*psmc6^hi3593Tg^*), genetic KOs define the developmental impact of decreased proteasome function. Importantly, loss of *psmb1* or *psmc6* results in widespread developmental abnormalities resembling EAE phenotypes, including growth restriction, abnormal craniofacial structure, neurodevelopmental defects, and failed hepatopancreas maturation. Furthermore, pharmacologic inhibition of chymotrypsin-like proteasome activity potentiates the teratogenic effects of EAE on craniofacial structure, the nervous system, and the endoderm. Our studies identify the proteasome as a target of EtOH exposure and signify that UPS disruptions contribute to craniofacial, neurological, and endodermal phenotypes in FASDs.

## Introduction

Prenatal alcohol exposure (PAE) can result in fetal alcohol spectrum disorders (FASDs), a common group of conditions affecting as many as 2%–5% of school children in the United States and Western Europe (1–5). FASDs range in severity and include fetal alcohol syndrome (FAS), partial FAS (pFAS), alcohol-related neurodevelopmental disorder (ARND), and alcohol-related birth defects (ARBD) (6, 7). Patients with FASDs exhibit a spectrum of phenotypes, including behavioral and cognitive disabilities, nerve and brain abnormalities, organ malformations, and craniofacial anomalies, thought to be related to disrupted development (6, 8). Despite the high incidence of FASDs throughout the world, the precise molecular mechanisms leading to FASDs have yet to be elucidated.

Although ethanol (EtOH) is a known teratogenic substance, the oxidative metabolism of EtOH by mother and fetus is also suggested to contribute mechanistically to FASDs (9–11). In the first stage of mammalian EtOH metabolism, coordinated action of several enzymes, including alcohol dehydrogenase (ADH) in the cytosol, cytochrome P450 2E1 (CYP2E1) in microsomes, and catalase in peroxisomes, convert EtOH into acetaldehyde (MeCHO) (12, 13). While the capacity of the fetus to metabolize EtOH is limited, enzymes ADH and CYP2E1 are present in the developing fetal liver and brain, respectively, by as early as 7–9 weeks gestation (14–16). The expression of these enzymes increases as the fetus ages, and importantly, MeCHO generated by maternal EtOH metabolism crosses the placenta and enters the fetal bloodstream (11, 15, 17, 18). In the next stages of EtOH metabolism, MeCHO is metabolized into acetate by aldehyde dehydrogenase (ALDH) (12, 13). The oxidative metabolism of EtOH results in the formation of MeCHO adducts, ROS, and an increased NADH/NAD^+^ ratio, all of which are destructive to cells (11–13). Excessive ROS can lead to oxidative stress–induced lipid peroxidation, resulting in toxic secondary products such as 4-hydroxynonenal (4-HNE) and malondialdehyde (MDA) (19). MeCHO, 4-HNE, and MDA form adducts with proteins, inactivating them and triggering their degradation (19–23). Given that protein adducts can impact protein homeostasis, we hypothesized that disruptions to the ubiquitin proteasome system (UPS) could serve as a significant contributing factor to the development of FASD-related phenotypes.

All cells rely on the UPS for the time-dependent degradation of short-lived regulatory proteins, the removal of damaged or misfolded proteins, and MHC class 1-restricted antigen processing (24). During intracellular protein degradation by the UPS, substrates are polyubiquitinated by ubiquitin ligases and targeted for degradation by the 26S proteasome, which is a complex consisting of a catalytically active 20S core and a 19S regulatory cap (25, 26). The 19S regulator, which contains ATPase subunits, restricts access to the 20S core and facilitates ATP-dependent substrate binding, deubiquitylation, and unfolding (26, 27). Substrates are then translocated to the 20S core, where they are degraded into small peptide fragments by subunits with chymotrypsin-like, trypsin-like, and caspase-like activities (26). In certain circumstances, several components of the constitutive 20S proteasome are replaced to form an “immunoproteasome” (28, 29). The 20S immunoproteasome can combine with an alternative regulator known as the PA28/11S, instead of or in addition to the 19S, to create a complex capable of facilitating the production of diverse MHC class I ligands (28, 29). Alternatively, the 20S immunoproteasome may regulate protein homeostasis in the context of oxidative stress, which could result from EtOH exposures (29–32). The role of specific proteasome components during development is poorly understood, especially as it pertains to the tissue-specific effects of component modulation.

Zebrafish larvae represent an ideal vertebrate model organism for FASDs because they are easily exposed to EtOH during development and recapitulate key features of the human syndrome, including developmental delay, short stature, craniofacial anomalies, cardiac defect, organ malformations, and behavioral alterations (33–38). Importantly, there is a high conservation between human and zebrafish genetics and physiology (39). In our model of embryonic alcohol exposure (EAE), larvae are exposed to 0.5%–1.0% EtOH after the completion of gastrulation (after 10 hours postfertilization [hpf]) until 5 days postfertilization (dpf) when larvae have developed mature organs. Studies conclude that the tissue concentration of EtOH in embryos ranges from 24% to 37% of external EtOH concentrations and decreases over time; therefore, 0.5%–1% EtOH exposures from 12 hpf to 5 dpf are in the range that is physiologically relevant for humans with chronic alcoholism throughout pregnancy (40–42).

Here, we utilize zebrafish to identify the UPS as a mechanistic target of EAE and demonstrate that disrupted protein homeostasis contributes to the development of FASD-related phenotypes. EAE dynamically altered gene expression of the 26S proteasome subunits and 11S regulator and led to increased ubiquitylated protein levels during development. Exposure to EtOH and MeCHO also impaired chymotrypsin-like proteasome peptidase activity in whole zebrafish larval lysates. Perturbing proteasome activity via pharmacologic proteasome inhibitors and genetic KO of 20S complex member *psmb1* and 19S ATPase *psmc6* led to growth deficits, neuronal apoptosis, craniofacial malformation, and endoderm defects, consistent with the phenotypes observed in EtOH-treated larvae. Finally, proteasome inhibition with chemical modulators bortezomib (BTZ) and MG132 potentiated the effects of EAE by increasing the incidence of Meckel’s cartilage abnormalities, causing excessive neuronal apoptosis and further reducing liver and pancreas size. Together, our data demonstrate that EtOH impairs protein degradation by the UPS during development and reveal that proteasome dysfunction via EtOH and its metabolites contributes to the teratogenicity of EtOH.

## Results

### Proteasome dysregulation is a consequence of EAE.

**Figure 1:**
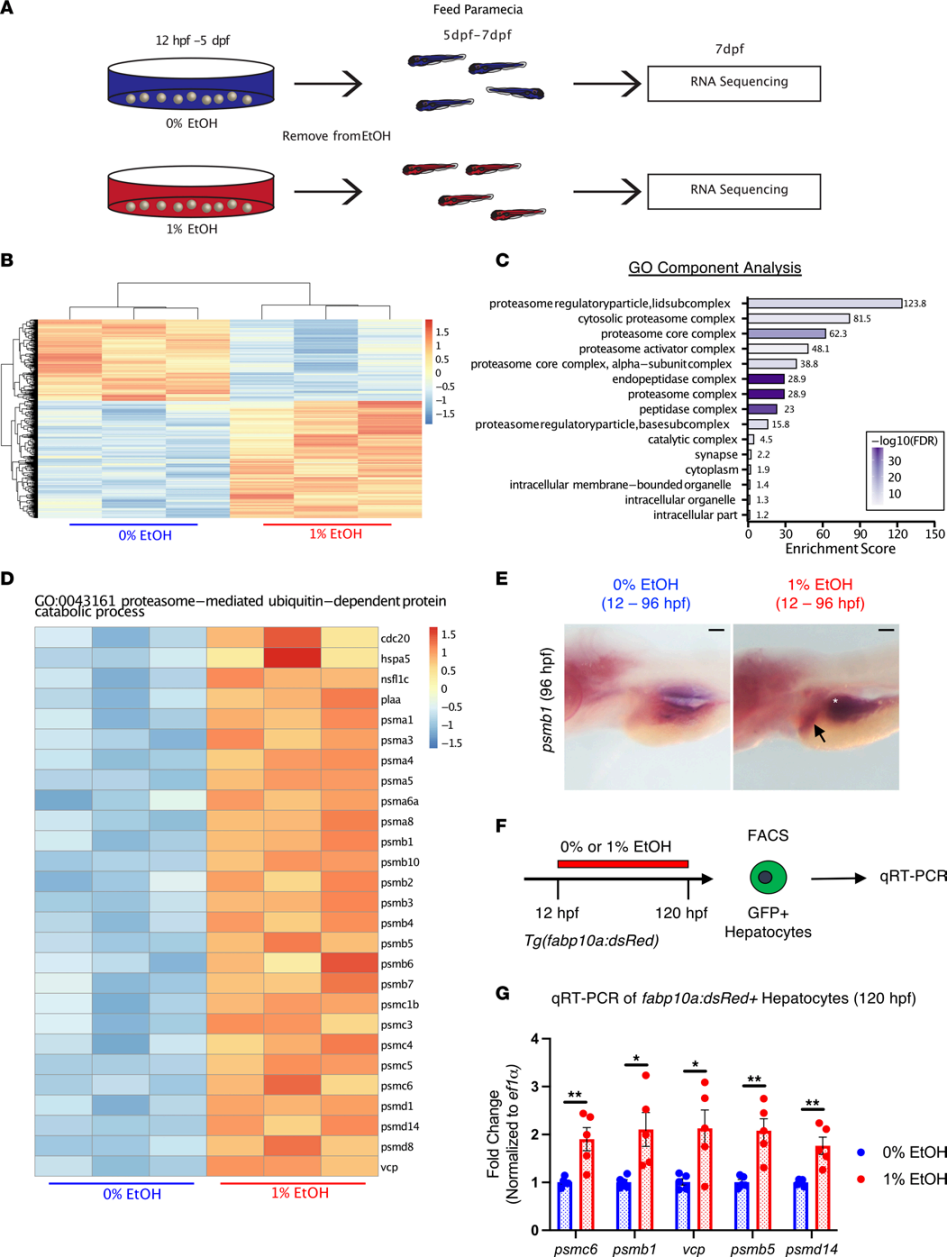
RNA-Seq identifies the ubiquitin proteasome system as a target of embryonic alcohol exposure. (**A**) Schematic of RNA-Seq performed on 7 dpf whole-larval extracts following 0% or 1% EtOH exposure (12 hpf–5 dpf). (**B**) Heatmap of significantly dysregulated genes (*P*_adj_ < 0.05; *n* = 1,603). (**C**) GOrilla GSEA identifies GO components enriched in the dysregulated gene set. (**D**) Heatmap of genes involved in proteasome-mediated, ubiquitin-dependent protein catabolism. Following EAE, these genes were significantly upregulated relative to controls. (**E**) ISH for *psmb1* following 0% or 1% EtOH exposure (12–96 hpf). At 96 hpf, *psmb1* expression is increased in the liver (black arrow) and intestine (white star). Scale bars: 100 μm. (**F**) Overview of the protocol for treatment and isolation of GFP^+^ hepatocytes via FACS sorting at 120 hpf. (**G**) ef1α-normalized qPCR of proteasome-related genes in GFP^+^ hepatocytes sorted by FACS (*P ≤ 0.05, **P ≤ 0.01, 2-sided *t* test; *n* = 5 per column). Data represent mean ± SEM.

To identify persistent transcriptional perturbations following EAE at postembryonic larval time points (72 or more hpf), we performed RNA-Seq on whole zebrafish larvae. Three independent groups of larvae from the AB background were exposed to 0% or 1% EtOH from 12 hpf to 5 dpf and subsequently incubated in EtOH-free embryo media containing paramecia from 5 to 7 dpf (Figure 1A). Allowing animals to recover for 48 hours enabled the identification of pathways that were dysregulated beyond the acute EtOH exposure period. Whole-organism RNA isolation and RNA-Seq were performed at 7 dpf (Figure 1A). In total, 1,603 genes were differentially regulated in the EAE group with a significance threshold of an adjusted *P* value (*P*_adj_) < 0.05 (Figure 1B and Supplemental Table 1; supplemental material available online with this article; https://doi.org/10.1172/jci.insight.156914DS1). Dysregulated genes were relevant to multiple critical developmental processes as indicated by Gene Set Enrichment Analysis (GSEA; Supplemental Figure 1A). GOrilla gene ontology (GO) analysis revealed alterations in pathways critical for normal protein homeostasis, including proteasomal ubiquitin-independent (*P* = 4.37 × 10^–22^) and ubiquitin-dependent (*P* = 4.72 × 10^–16^) protein catabolism, protein glycosylation (*P* = 2.75 × 10^–4^), and protein folding (*P* = 5.35 × 10^–4^; Figure 1C and Supplemental Table 2). The proteasome complex, composed of many conserved subunits, was the most significantly enriched GO component (*P* = 4.15 × 10^–41^; Figure 1C and Supplemental Table 3). Genes relevant to proteasome function, including those that encode members of the 20S proteasome core complex, the 19S regulatory particle, and the 11S/P28 regulatory complex, were upregulated in EAE larvae (Figure 1, C and D; Supplemental Figure 1B; and Supplemental Table 3). The most significantly upregulated proteasome subunits included *psmc1b*, *psme2*, *psma4*, *psma5*, *psmd11b*, *psmb1*, *psmb4*, *psmb3*, *psmc4*, *psmd3*, and *psmc6* (Figure 1D, Supplemental Figure 1B, and Supplemental Table 1). Similarly, expression of ubiquitin-like modifier activating enzyme 7 (*uba7*), ER stress–inducible gene binding immunoglobulin protein (*hspa5/bip*), and ATPase valosin-containing protein (*vcp/p97*), which facilitates the degradation of polyubiquitinated proteins, were significantly upregulated in the EAE group (Figure 1D and Supplemental Table 1).

To evaluate the spatiotemporal impact of EtOH on proteasome gene expression throughout earlier stages of development, we utilized in situ hybridization (ISH) and quantitative PCR (qPCR), focusing ISH on genes for which genetic mutants are available. By ISH, 1% EtOH exposure (12–96 hpf) increased *psmb1* signal in the developing gut tube and liver, suggesting that these endoderm derivatives are a site for EtOH-induced proteasome upregulation (Figure 1E). To confirm whether other proteasome genes were similarly upregulated in the liver, dsRed^+^ hepatocytes from *Tg(fabp10a:dsRed; elastase:GFP)* reporter embryos treated with 0% or 1% EtOH (12–120 hpf) were isolated by FACS and evaluated by qPCR (Figure 1F). Embryos that were 120 hpf were utilized because EtOH exposure reduces liver size and the 120 hpf time point provides sufficient differentiated cells for analysis (43). All examined proteasome-related genes showed increased expression in EtOH-exposed hepatocytes, including *psmb1*, *psmc6*, *vcp*, *psmb5*, and *psmd14* (Figure 1G). This finding corroborates the RNA-Seq results and demonstrates that proteasome upregulation occurs in EAE-exposed liver and gut tube.

Next, we examined the impact of EAE on proteasome gene expression in other tissues at earlier developmental time points (30–96 hpf) to capture the development of the head and cranial neural crest cells (30 hpf), the hepatopancreatic buds within the developing endoderm (50 hpf), and liver and pancreas differentiation (96 hpf). EAE (1% EtOH, 12–30 hpf) significantly increased the expression of *psmc6*, but not other proteasome-related genes, by qPCR in pooled surgically isolated heads at 30 hpf, suggesting proteasome dysregulation in the head during early development (Supplemental Figure 1C). We also performed qPCR on whole homogenized embryos following EAE to determine whether 11S/PA28 regulator components *psme1* and *psme2*, identified in the RNA-Seq results, were also dysregulated. The 1% EtOH exposure beginning at 12 hpf caused significant upregulation of *psme1* at 30 and 50 hpf, followed by significant downregulation of both *psme1* and *psme2* by 96 hpf (Supplemental Figure 1D). These data demonstrate that EtOH dynamically affects the expression of proteasome components throughout development in a tissue-specific manner and suggests that these changes may contribute to FASD-related pathologies.

### EtOH impairs chymotrypsin-like proteasome peptidase activity in a cell type–specific manner.

**Figure 2:**
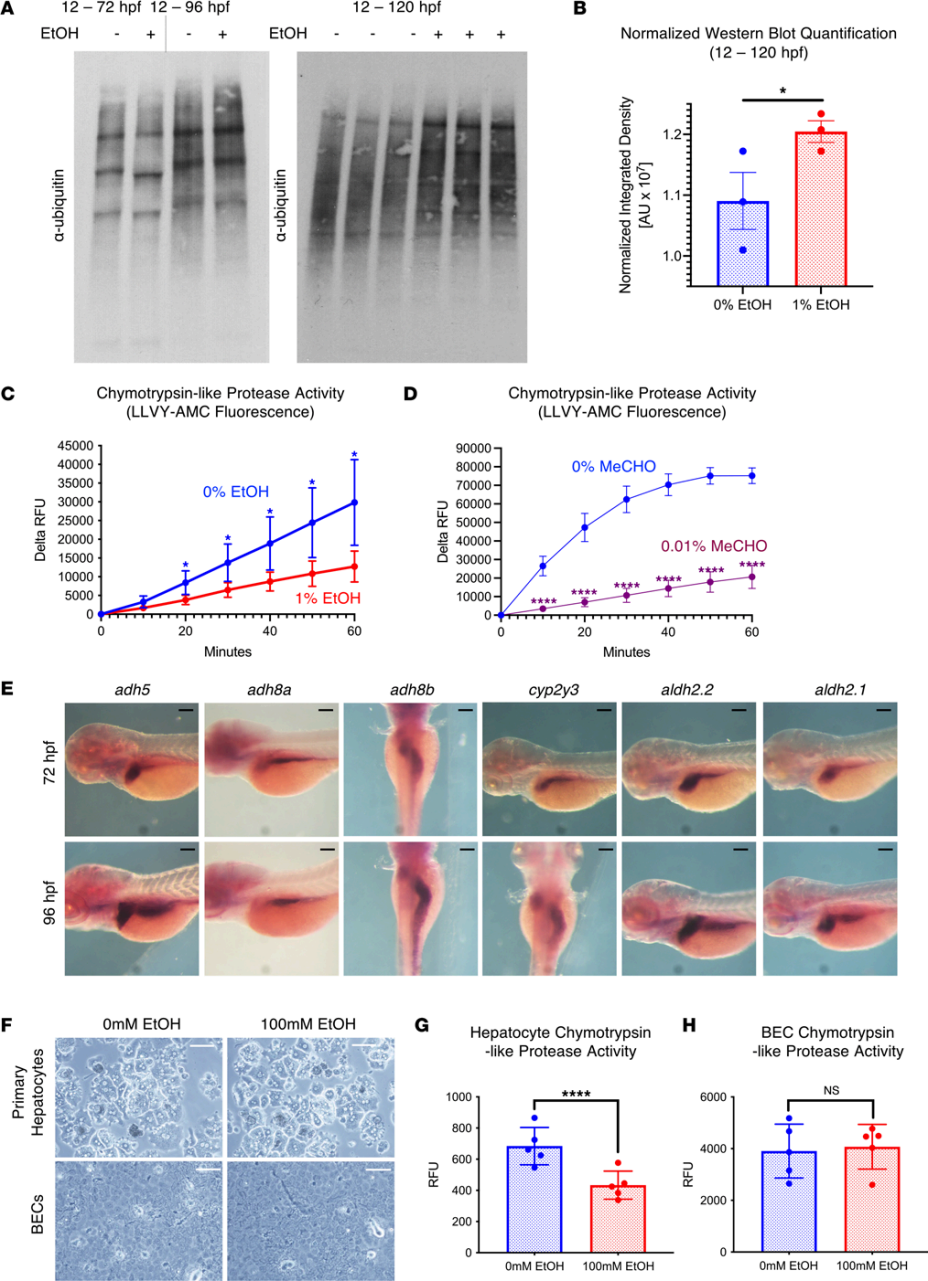
EtOH modulates the ubiquitin proteasome system and chymotrypsin-like proteasome peptidase activity in a cell type–specific manner. (**A**) Western blot analysis of ubiquitylated protein and the 20S proteasome after exposure to 0% or 1% EtOH. See Supplemental Figure 2A for loading controls. (**B**) ImageJ quantification of normalized ubiquitylated protein levels in embryos treated with 0% and 1% EtOH (12–120 hpf; 1-sided unpaired *t* test, *P ≤ 0.05; *n* = 3). (**C** and **D**) Proteasome activity assay in protein extracts of from whole homogenized 5 dpf larvae. Chymotrypsin-like proteasome activity is impaired by 1% EtOH exposure (12 hpf–5 dpf) and 0.01% MeCHO exposure (104–120 hpf; *P < 0.05, ****P ≤ 0.0001, 2-sided *t* test per time point; *n* = 5). (**E**) Time course ISH for EtOH and MeCHO metabolism genes. For most genes, expression after 72 hpf is noted in the liver and intestine. (**F**) Confocal imaging of 2D-plated hepatic and biliary epithelial cell (BEC) organoids after 24 hours of treatment with 0 or 100mM EtOH. (**G** and **H**) Exposure to 100 mM EtOH impairs chymotrypsin-like proteasome activity in 2D hepatic organoids but not BECs (****P < 0.0001, 2-sided *t* test; *n* = 5). Scale bars: 100 μm. For **B**, **G**, and **H**, data represent mean ± SEM. For **C** and **D**, data represent mean ± SD.

EtOH is thought to disrupt protein homeostasis via several mechanisms, including by inhibiting proteasome activity, inducing translation error, and impairing ER function (44–48). We next sought to determine the impact of EtOH exposure (12 hpf collection point) on protein homeostasis in developing larvae following the formation of the liver (72–120 or more hpf) by performing Western blot (WB) for ubiquitin (P4D1). Ubiquitylated protein levels were not elevated by EAE at either 72 hpf or 96 hpf; however, WB analysis demonstrated an increase in ubiquitylated protein load at 120 hpf following EAE (Figure 2, A and B, and Supplemental Figure 2A). Changes in ubiquitylation at later stages (120 hpf) but not earlier stages (72–96 hpf) may indicate that time is required for protein accumulation to occur, that EtOH-induced proteasome inhibition only occurs at later stages when EtOH metabolism is underway, or that certain organs, such as the liver and pancreas, must represent a high enough fraction of the sample to detect the ubiquitylated protein accumulation (Figure 2A). Coupled with the RNA-Seq data, the presence of increased ubiquitylated protein is consistent with proteotoxic stress or impaired protein degradation.

Although the exact mechanisms responsible for EtOH-induced proteasome inhibition are unresolved, toxic metabolites resulting from EtOH metabolism can inhibit the proteasome by forming damaging adducts (12, 49, 50). To test our hypothesis that EAE impairs proteasome function when elevated ubiquitylated protein levels are detected by WB, we evaluated whether EtOH is capable of inhibiting chymotrypsin-like and caspase-like proteasome peptidase activity in whole-larval extracts after treatment with 0% EtOH or 1% EtOH (12–120 hpf) using an AMC-based assay. The 1% EtOH exposure significantly reduced chymotrypsin-like (LLVY-AMC peptide) proteasome peptidase activity but not caspase-like activity (Z-LLE-AMC peptide) at 120 hpf (Figure 2C and Supplemental Figure 2B). Embryos were also treated with 0.01% MeCHO from 104 hpf to 120 hpf, and proteasome peptidase activity was evaluated in whole-larval extracts. MeCHO is significantly more toxic than EtOH, and the 0.01% concentration used was determined by a survival assay in which embryos were exposed to a dose-curve of concentrations from 0% to 0.5% (data not shown). The 0.01% MeCHO was a concentration that allowed animal survival while producing visible developmental abnormalities, and it is consistent with previous literature (51). Using this concentration, MeCHO similarly significantly reduced proteasome peptidase activity (Figure 2D). These data indicate that exposure to EtOH reduces proteasome peptidase activity during development and that this inhibition may be mediated by toxic metabolites such as MeCHO.

To better define the spatiotemporal dynamics of EtOH metabolism in developing embryos, we examined gene expression of key enzymes involved in this process. At 48 hpf, *adh5*, *adh8a*, *adh8b*, and *aldh2.2* were each diffusely expressed throughout the brain, with *adh5*, *cyp2y3*, and *aldh2.2* showing additional expression in the hepatopancreatic progenitors, which will form the liver and pancreas (Supplemental Figure 2C). By 72 hpf, all genes examined were strongly expressed in the liver and intestinal tract (Figure 2E). This expression pattern is consistent with a role for brain and endodermal organs as primary sites of EtOH and MeCHO metabolism in developing larvae and implies these cell populations may be prone to UPS disruptions during development.

The liver contains multiple cell types with different capacities for EtOH metabolism, including hepatocytes and biliary epithelial cells (BECs). To determine whether there are cell type–specific effects of EtOH on the UPS, we examined chymotrypsin-like proteasome peptidase activity in lysates from organoid cultures of murine primary hepatocytes and BECs. Both cell types were exposed to 100 mM EtOH in vitro (with limited impact on cell number or morphology observed) and were evaluated for chymotrypsin-like proteasome activity (Figure 2F). EtOH significantly impaired proteasome peptidase activity in hepatocytes, but not BECs (Figure 2, G and H). These data further suggest that EtOH metabolism, characteristic of hepatocytes and not BECs, may specifically contribute to altered protein homeostasis in the context EtOH-induced tissue injury.

To assess whether EtOH exposure has a conserved impact on the UPS in the adult zebrafish liver, and to determine which components are transcriptionally affected, we examined hepatic tissue from age-matched 1-year-old zebrafish (gut:GFP) that were subjected to 0% EtOH or 0.9% EtOH for 24 hours, which models a binge-drinking episode (Supplemental Figure 2D). Livers were surgically removed, and qPCR was performed to examine the expression of a subset of genes relevant to protein catabolism (Supplemental Figure 2D). EtOH exposure resulted in a significant increase in the expression of the 19S ATPase *psmc6* and chaperone *vcp* (Supplemental Figure 2E), suggesting that, in zebrafish, EtOH can perturb the UPS in mature hepatic tissue (Figure 1, F and G).

### Proteasome inhibition and misfolded protein accumulation mimic transcriptional signatures of EtOH exposure in zebrafish.

**Figure 3:**
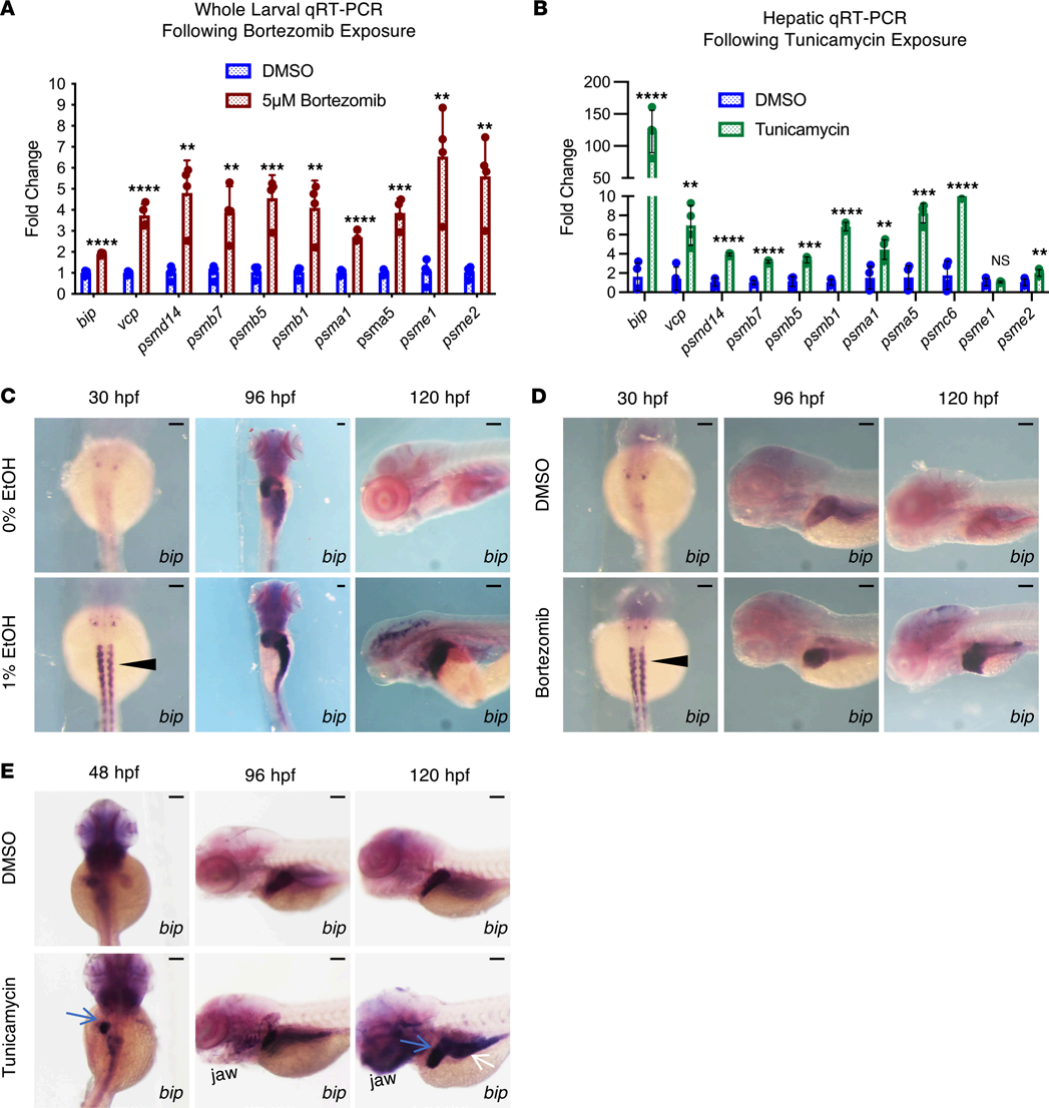
Disruptions to the ubiquitin proteasome system trigger proteasome compensation and produce transcriptional signatures similar to EAE. (**A** and **B**) qPCR analysis of 5 dpf whole-larval extracts from DMSO and BTZ treatment (16 hours) (**A**) and 60 dpf whole-liver extracts from DMSO and Tm treatment (12 hours). Expression was normalized to *ef1α*. Discovery was determined using the 2-stage linear step-up procedure of Benjamini, Krieger, and Yekutieli, (**P ≤ 0.01, ***P ≤ 0.001, ****P ≤ 0.0001; each gene was analyzed individually using an unpaired 2-tailed *t* test). (**C**) ISH for *bip* following 0% and 1% EtOH (exposure window 12 hpf–5 dpf) treatment. EAE increases *bip* expression in the somites at 30 hpf (black arrowhead), and the liver, intestine, and brain from 96 to 120 hpf. (**D**) ISH for *bip* following DMSO and BTZ (exposure window 12 hpf–5 dpf) treatment. BTZ increases *bip* expression in the somites at 30 hpf (black arrowhead), and the liver, intestine, brain, and pancreas from 96 to 120 hpf. (**E**) ISH for *bip* in DMSO- and Tm-treated embryos. Tm increases *bip* expression in the endoderm, including the hepatoblasts (48 hpf, blue arrow), liver (120 hpf, blue arrow), and gut tube (120 hpf, white arrow). Scale bars: 100 μm. Data represent mean ± SD.

We next assessed whether proteasome inhibition caused by BTZ or protein misfolding induced by tunicamycin (Tm) could replicate the broad proteasome-related gene upregulation observed in EAE larvae at 7 dpf. BTZ binds to the catalytic site on the β5 subunit, inactivating the chymotrypsin-like activity of the proteasome, which is also impaired by EtOH and MeCHO exposure (Figure 2, C, D, and G). Zebrafish larvae were treated with 5 μM of potent proteasome inhibitor BTZ from 4.5 to 5 dpf, and acute gene expression changes were evaluated. Expression of 26S complex members (*psmd14*, *psmb7*, *psmb5*, *psmb1*, *psma1*, *psmc5*, *psmc6*), 11S complex members (*psme1*, *psme2*), and chaperones (*bip*, *vcp*) were significantly induced by BTZ in whole-larval extracts (Figure 3A). To evaluate whether protein misfolding and related ER stress could also impact the expression of proteasome-related genes in zebrafish, juveniles (~ 60 dpf) were treated with the N-linked glycosylation inhibitor Tm for 12 hours, which is known to cause unfolded protein stress in the liver (52). Livers were extracted for tissue-specific RNA analysis, and except for *psme1*, each UPS component examined was found to be significantly induced by Tm in isolated hepatocytes (Figure 3B). These findings suggest that proteasome inhibition and increased protein misfolding can trigger compensatory proteasome upregulation and may represent underlying mechanisms in EtOH-induced dysregulation of protein homeostasis.

Binding immunoglobulin protein (*bip/hspa5*) is a molecular chaperone located in the ER that regulates protein folding, protein import to the ER, initiation of the unfolded protein response (UPR), and ER-associated degradation (53). Exposure to EtOH, BTZ, or Tm significantly induced *bip* (Figure 1D and Figure 3, A and B) (54). To identify cell types that potentially experience UPR in response to each stressor, we characterized *bip* expression in developing embryos by ISH. The 1% EtOH and 5 μM BTZ exposure (from initiation at 12 hpf to 5 dpf) both resulted in excess *bip* expression in somites (30 hpf), liver and gastrointestinal (GI) tract (96–120 hpf), and brain (120 hpf) relative to controls (Figure 3, C and D). Tm induced *bip* expression in the liver, the GI tract, and the lower jaw (Figure 3E) (55). These data indicate a significant spatial overlap in *bip* induction in embryos exposed to EtOH, BTZ, or Tm. Importantly, the induction of *bip* by EtOH more closely resembled the expression pattern observed with proteasome inhibition (BTZ) than with generalized protein misfolding (Tm). While not excluding additional or alternative responses in select tissues, together, these observations demonstrate that EtOH-associated alterations in UPS-related genes can result from proteasome inhibition and downstream misfolded protein accumulation.

### Disruption of proteasome function causes developmental disorder in zebrafish.

**Figure 4:**
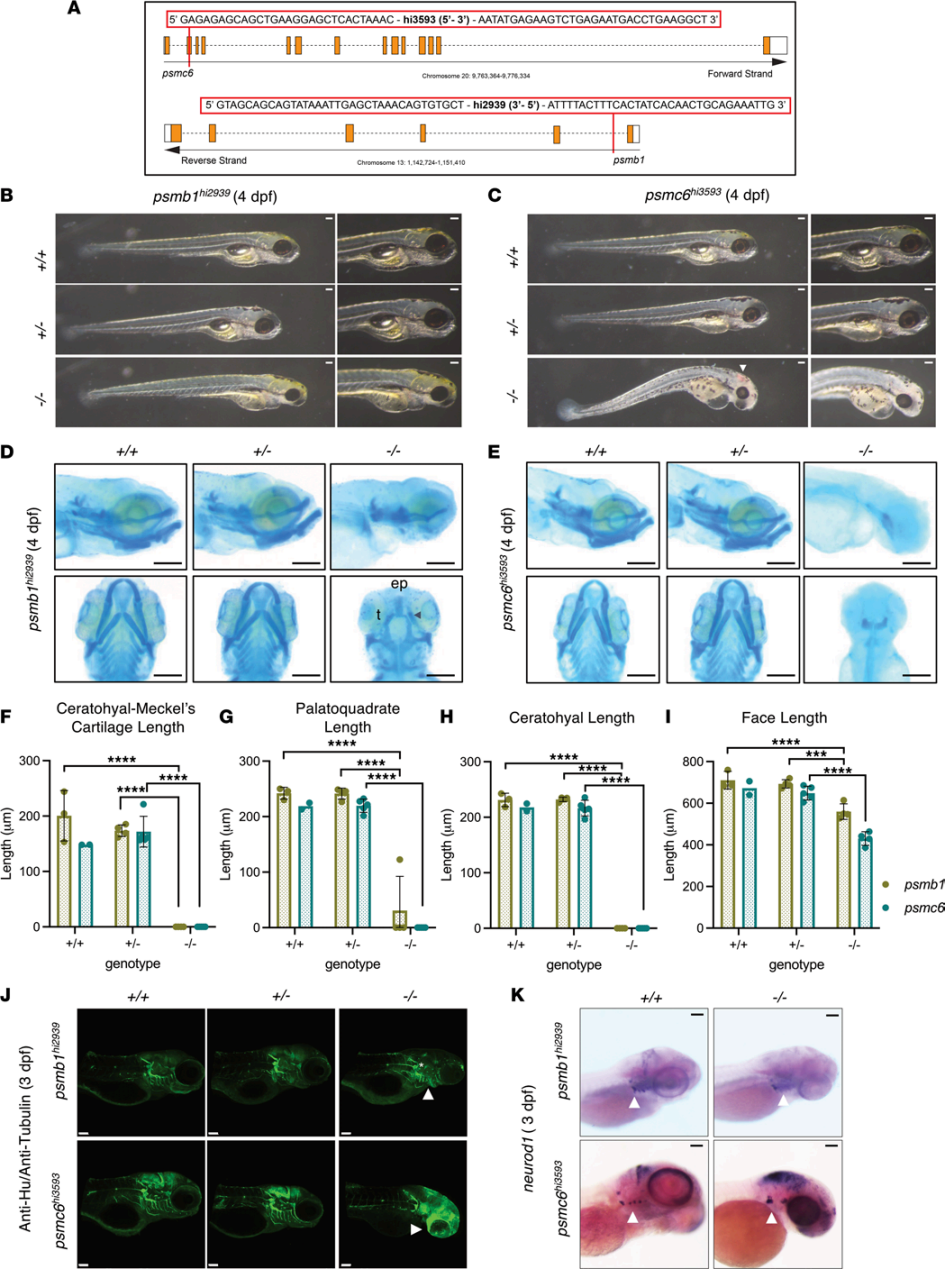
Psmb1 and Psmc6 are required for craniofacial and nervous system development. (**A**) Map of the transgenic insertion sites for *psmb1^hi2939^* and *psmc6^hi3593^* mutants. (**B** and **C**) Widefield imaging of *psmb1^hi2939^* and *psmc6^hi3593^* mutants. Both have craniofacial malformations, and *psmc6^–/–^* larvae have cardiac edema and blood pooling in the brain (**C**, white arrowhead). (**D** and **E**) Widefield imaging of Alcian blue–stained larvae. (**D**) *psmb1^–/–^* larvae (4 dpf) lack cartilages contributing to the lower jaw and have a reduced ethmoid plate (ep) and abnormal trabecula (t). Cartilage remnants (arrowhead) from the lower jaw appear in a subset of homozygotes. (**E**) *psmc6^–/–^* lack most cranial cartilage. (**F**–**I**) Measurements of craniofacial features obtained from ImageJ analysis of Alcian blue–stained larvae (4 dpf; ***P ≤ 0.001, ****P ≤ 0.0001, ordinary 1-way ANOVA with Dunnett’s multiple-comparison test following an observation of a difference in measurements between +/+ and –/– embryos; *psmb1^+/+^*, *n* = 3; *psmb1^+/–^*, *n* = 4; *psmb1^–/–^*, *n* = 4; *psmc6^+/+^*, *n* = 2; *psmc6^+/–^*, *n* = 5; and *psmc6^–/–^*, *n* = 5). (**J**) Anti-Hu/Anti-tubulin IHC at 72 hpf. *psmb1^–/–^* and *psmc6^–/–^* have abnormal brain structure and cranial ganglia (white star) and nerve development (white arrowheads). (**K**) ISH for *neurod1* at 72 hpf. *psmb1^–/–^* and *psmc6^–/–^* have abnormal and missing vagal/cranial ganglia (white arrowheads). Scale bars: 100 μm. Data shown represent mean ± SD.

To directly interrogate the role of the proteasome in organ development, we evaluated the impact of genetic loss in *psmc6^hi3593^* and *psmb1^hi2939^* mutants generated in a previous insertional mutagenesis screen (Figure 4A) (56–59). Both *psmb1* and *psmc6* are expressed in the developing brain, eye, and GI tract, as detected by ISH (Supplemental Figure 3A). Importantly, homozygous *psmb1^hi2939^* and *psmc6^hi3593^* mutants had increases in ubiquitylated protein relative to WT and heterozygous embryo pools by WB, confirming disruptions in the UPS (Supplemental Figure 3B). Homozygotes displayed developmental abnormalities, including absent swim bladder, craniofacial anomaly, and reduced eye size at 4 dpf (Figure 4, B and C). The *psmc6^–/–^* mutants were more severely affected, with demonstrated growth restriction, additional cardiac edema, and brain hemorrhage indicative of cardiovascular abnormalities (Figure 4C). Between 3 and 4 dpf, tissues within *psmc6^–/–^* larvae became dusky, and by 4.5 dpf, heartbeat ceased (Figure 4C); *psmb1^–/–^* larvae were uniformly embryonic lethal by 5 dpf. At 24 hpf, homozygous *psmb1^hi2939^* and *psmc6^hi3593^* mutants were indistinguishable from WT siblings, and only *psmc6* homozygotes had visible alterations at 48 hpf in the form of reduced eye size (Supplemental Figure 3, C and D).

FAS and pFAS include craniofacial anomalies as diagnostic criteria (6). Craniofacial alterations are also documented in EtOH-exposed zebrafish embryos (35, 60). We utilized Alcian blue staining to characterize cranial cartilage development in *psmb1^hi2939^* and *psmc6^hi3593^* mutants at 4 dpf. Heterozygotes from both mutant lines were normal, whereas homozygotes exhibited substantial craniofacial defects (Figure 4, D and E). The *psmb1^–/–^* larvae had absent lower jaw structures, reduced ethmoid plate, and trabeculae defects (Figure 4D). The *psmc6^–/–^* larvae lacked most cranial cartilage; they had significant reductions in the palate and loss of the lower jaw (Figure 4E). Measurements of the distance (illustrated in Supplemental Figure 3E) between the ceratohyal and Meckel’s cartilage, palatoquadrate length, ceratohyal length, and overall face length were unaffected in *psmb1* and *psmc6* heterozygotes; however, each of these measures were decreased in homozygotes (Figure 4, F–I). The decrease in face length in the *psmc6* homozygotes may be partly explained by generalized developmental delay and growth deficiency. However, *psmb1* mutants had a longer body length than WT siblings with a concomitant significant reduction in face length/body length ratio (Supplemental Figure 3, F–H). This indicates that, for *psmb1* mutants, reduction in face length is not explained by generalized growth retardation.

In addition to craniofacial phenotypes, *psmb1^hi2939^* and *psmc6^hi3593^* homozygotes had neurological abnormalities. IHC for HuC/D, which marks neuronal cell bodies, and acetylated tubulin, which stains axons, showed that *psmb1^–/–^* larvae had reduced cranial ganglia and improper migration of the cranial nerves at 3 dpf (Figure 4J, top panel, white arrow). In particular, the vagal ganglia, branches of the vagal nerve, and the glossopharyngeal nerve were reduced (Figure 4J, top panel, white star) (61). Similarly, *psmc6^–/–^* larvae had missing and improperly positioned cranial ganglia and a lack of cranial nerve innervation of the pharyngeal arch derivatives (Figure 4J, bottom panel, white arrow). Likewise, in situ hybridization for neuronal differentiation 1 (*neurod1*) confirmed abnormal cranial ganglia in both mutants (Figure 4K, white arrows). Notably, *psmb1^–/–^* larvae also had dramatic upregulation of chaperone *vcp* and mild elevation of *bip* in the lower jaw (white arrows) and brain (black arrows), indicating that these cell types may be experiencing disproportionate amounts of proteotoxic stress (Supplemental Figure 3, I and J).

### Proteasome inhibition potentiates EtOH toxicity and exacerbates EtOH-induced organ malformations.

**Figure 5:**
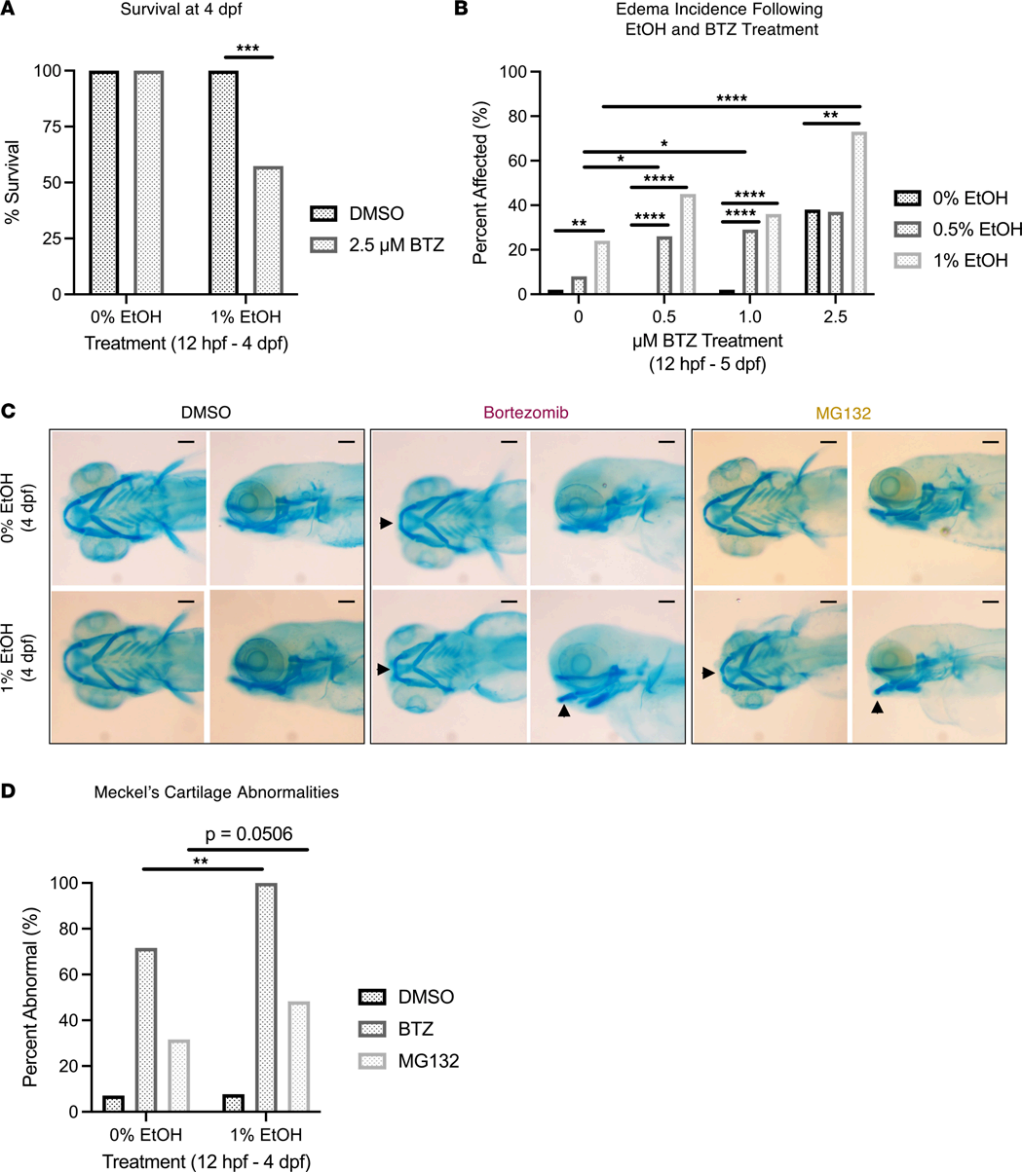
Proteasome inhibition potentiates EtOH toxicity and exacerbates EtOH-induced craniofacial anomalies. (**A**) Larval survival at 4 dpf after treatment with 1% EtOH (12 hpf–4 dpf) or 2.5 μM BTZ. Survival significantly decreases following exposure to 1% EtOH and BTZ in combination. (**B**) Edema prevalence in 5 dpf larvae exposed to a EtOH, BTZ, or both in increasing concentrations. For **A** and **B**, *P* values determined by a 2-sided Fischer’s exact test (*P < 0.05, **P ≤ 0.01, ****P ≤ 0.0001). (**C**) Alcian blue staining of 0% and 1% EtOH-treated (12 hpf–4 dpf) larvae in the presence of DMSO, BTZ, and MG132. BTZ and MG132, especially in the context of 1% EtOH exposure, induced Meckel’s cartilage malformations (black arrows). (**D**) Quantification of the percentage of affected individuals with Meckel’s cartilage abnormalities from each condition (**P < 0.01, 2-sided Fischer’s exact test). BTZ concentration = 2.5 μM (12 hpf–4 dpf). MG132 concentration = 5 μM (12 hpf–4 dpf). Scale bars: 100 μm. For **D**, percentages are based on *n* > 30 per group. See Supplemental Tables 4 and 5 for raw data and statistics related to **A** and **B**.

Our data indicate that EtOH impairs proteasome function and that preexisting reductions in proteasome activity may, therefore, sensitize embryos to EAE. To determine whether pharmacological inhibition of proteasome function increases the susceptibility to EtOH-induced developmental defects, embryos were treated with proteasome inhibitor BTZ (0.5–5.0 μM) or MG132 (5 μM), a synthetic peptide aldehyde that acts as an inhibitor of chymotrypsin-like proteasome activity, in the presence or absence of EtOH (0%, 0.5%, 1.0%) beginning at 12 hpf. Larvae cotreated with BTZ and EtOH had significantly reduced survival at 4 dpf relative to larvae exposed to either EtOH or BTZ alone (Figure 5A and Supplemental Table 4). Furthermore, surviving larvae treated with EtOH and BTZ had a dose-dependent increase in edema incidence at 5 dpf relative to larvae treated with EtOH or BTZ alone (Figure 5B and Supplemental Table 5). Larvae cotreated with EtOH and MG132 had similar increases in mortality and edema (data not shown). These findings demonstrate that proteasome inhibition increases the lethal impact and teratogenicity of EtOH and further highlights that proteasome function is protective against EAE.

We next evaluated whether proteasome inhibition combined with EAE exacerbates the presence of craniofacial phenotypes. *psmb1* and *psmc6* heterozygotes showed little evidence of haploinsufficiency. Consistent with a lack of gene dosage effect, craniofacial development in heterozygotes was not differentially sensitized to EtOH-induced injury (treated from 12 hpf to 4 dpf) compared with sibling-matched WT larvae (Supplemental Figure 4, A–J). Furthermore, *psmb1^+/–^* were not significantly more sensitized to BTZ-induced craniofacial abnormalities, such as reduced ceratohyal to Meckel’s cartilage length, than WT siblings (Supplemental Figure 4K). Therefore, we turned to pharmacologic proteasome inhibition. Embryos were treated with BTZ and MG132 in the presence and absence of EtOH, and cartilage was evaluated. Proteasome inhibitors disrupted craniofacial development and induced mild Meckel’s cartilage abnormalities, revealing squaring of Meckel’s cartilage (Figure 5C). Coexposure with 1% EtOH and BTZ significantly increased the severity and incidence rate of Meckel’s cartilage malformations relative to proteasome inhibitor treatment alone (Figure 5, C and D). Furthermore, 1% EtOH plus BTZ or MG132 also significantly reduced head length, ceratohyal length, ceratohyal to palatoquadrate length, and palatoquadrate length (Supplemental Figure 4, L–P). Cotreatment with EtOH and BTZ increased ceratohyal angle relative to BTZ or EtOH alone (Supplemental Figure 4P). These findings demonstrate that inhibition of proteasome catalytic activity may sensitize embryos to craniofacial malformations induced by EAE.

### Increased apoptosis contributes to craniofacial, CNS, and PNS anomalies in proteasome mutants.

**Figure 6:**
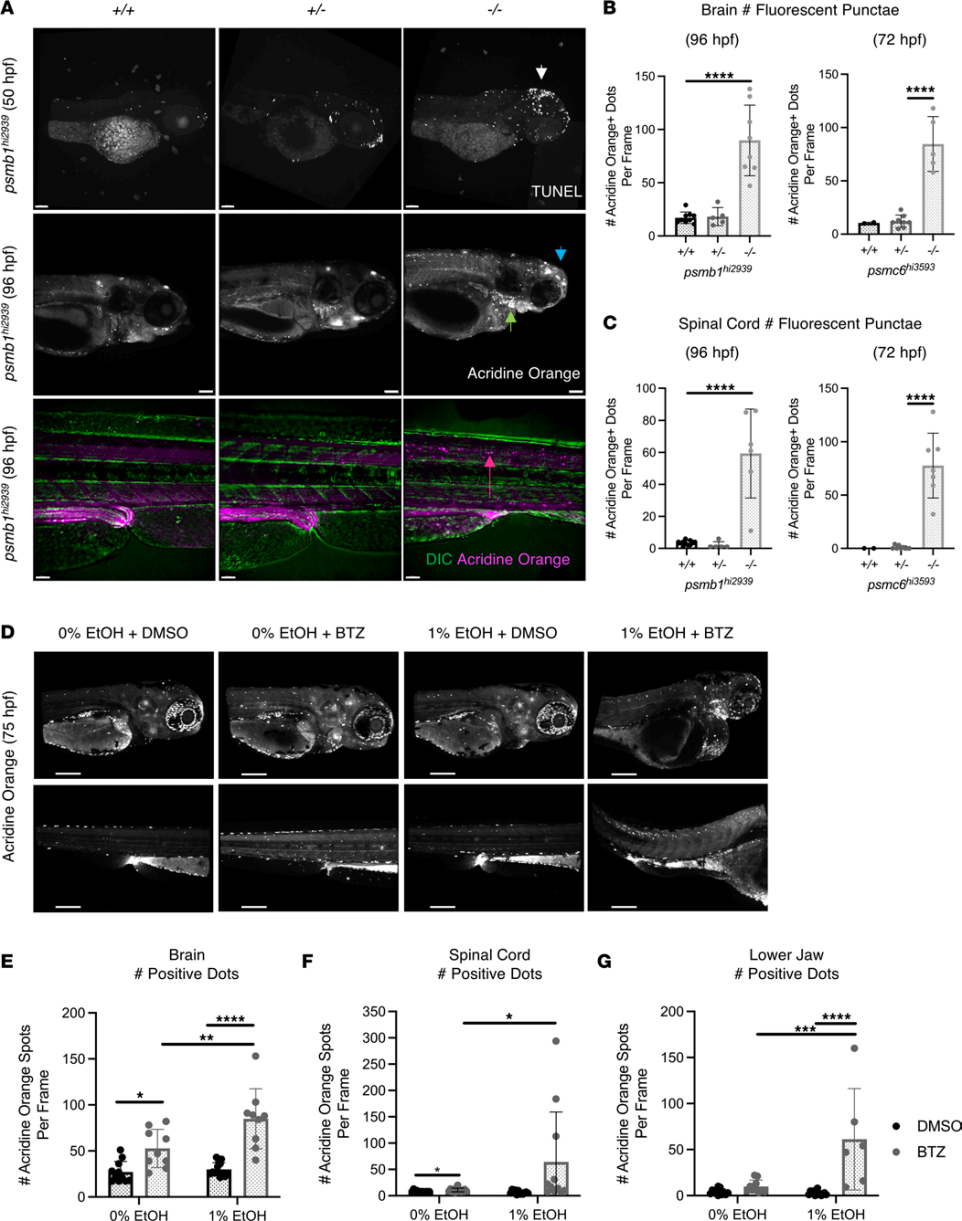
*psmb1* and *psmc6* are required for cell survival in the developing brain, spinal cord, and pharyngeal arches. (**A**) Confocal image analysis of acridine orange–stained (AO-stained) and TUNEL-stained *psmb1^hi2939^* (50 hpf, 72 hpf, 96 hpf) mutants reveals increased apoptosis in the brain (top and middle rows, blue and white arrows) and spinal cord (bottom row, pink arrow). *psmb1^–/–^* have increased labeling in the pharyngeal arch area (middle row, green arrow). (**B** and **C**) ImageJ (NIH) quantification of fluorescent punctae in the brain and spinal cord of mutants following AO staining (****P ≤ 0.0001, unpaired 2-tailed *t* test; for +/+ vs. +/–, *P* > 0.05). For columns left to right, *n* = 10, 5, 6, 2, 8, 5 (**B**) and *n* = 9, 5, 8, 2, 9, 7 (**C**). (**D**–**G**) Confocal imaging and quantification of AO-stained larvae (75 hpf). Exposure to BTZ (2 μM, 12–75 hpf) alone resulted in significantly increased staining in the brain. Cotreatment with EtOH and BTZ increased the number of positive dots in the brain, spinal cord, and pharyngeal arch area relative to EtOH and BTZ alone. *P < 0.05, **P < 0.01, ***P < 0.001, ****P ≤ 0.0001, 2-way ANOVA with Tukey’s multiple-comparison test. From left, column sample *n* = 11, 9, 13, 9 (**E**); *n* = 12, 13, 12, 11 (**F**); and *n* = 12, 10, 13, 6 (**G**). Scale bars: 100 μm. Data represent mean ± SD.

Alterations in the UPS are known to mediate neuronal apoptosis (62). We next evaluated the impact of *psmb1* and *psmc6* KO on apoptosis throughout a developmental time series using fluorescent acridine orange (AO) and TUNEL staining. Cell death in the CNS of *psmb1^–/–^* embryos was detected by TUNEL staining; it began prior to 50 hpf (Figure 6A) and continued until embryonic lethality. Homozygous mutants of both genotypes, but not heterozygotes, had significant increases in fluorescent punctae in their forebrain, hindbrain, and spinal cord by AO staining (Figure 6, A–C, and Supplemental Figure 5, A–C). These data reveal that proteasome inhibition causes substantial cell death in the nervous system, resulting in widespread neurological abnormalities. Importantly, *psmb1^–/–^* larvae also exhibited intense AO staining in the pharyngeal arch derivatives, indicating that increased cell death or cellular stress resulting in excess lysosomes (stained by AO) in this region may contribute to the craniofacial abnormalities observed (Figure 6A). PAE has been previously shown to induce neuronal apoptosis (63–65). Overlapping phenotypes suggest that proteasome inhibition may serve as a contributing mechanism for neuronal apoptosis following EAE.

We next tested whether *psmb1* or *psmc6* heterozygotes had increased apoptosis in response to BTZ or EtOH exposure relative to WT siblings. The *psmb1* heterozygotes showed no significant increase in TUNEL^+^ brain cells relative to WT siblings following BTZ treatment (Supplemental Figure 5, D and E). This demonstrates that heterozygotes are unlikely to have significant deficits in proteasome function, which could be explained by the fact that a single gene copy is sufficient to produce enough of the proteasome component or by the fact that the mutated component is not rate limiting in proteasome complex assembly. Furthermore, shorter exposure to 1% EtOH (48–78 hpf) to capture resulting cell death significantly increased apoptosis in *psmc6^+/+^* and *psmc6^+/–^* siblings; however, *psmc6^+/–^* heterozygotes also showed no evidence of increased EtOH sensitivity (Supplemental Figure 5, F and G). In the absence of evidence of haploinsufficiency in heterozygous mutants, we determined whether partial inhibition of chymotrypsin-like proteasome activity with BTZ was sufficient to sensitize neurons to EtOH-induced cell death using AO stain. Larvae (75 hpf) treated with 2 μM BTZ (12–75 hpf) had a significantly increased number of fluorescent punctae in the brain and spinal cord relative to DMSO treatment, whereas 1% EtOH alone (12–75 hpf) had a limited effect on cell survival during this treatment window (Figure 6, D–F). Treatment with 2 μM BTZ alone did not significantly increase AO staining in the lower jaw, likely because pharmacological inhibition at that dosage is less complete than genetic ablation (Figure 6G). However, cotreatment of embryos with EtOH and BTZ resulted in dramatic increases in apoptosis in the brain, spinal cord, and lower jaw, which is consistent with what is observed in homozygous proteasome mutants (Figure 6, D–G). Two-way ANOVA revealed a significant interaction (*P* < 0.05) between drug treatment and EtOH exposure in each case, indicating that EtOH interacts with the proteasome to promote cell death.

### Proteasome function is required for hepatopancreas development.

**Figure 7:**
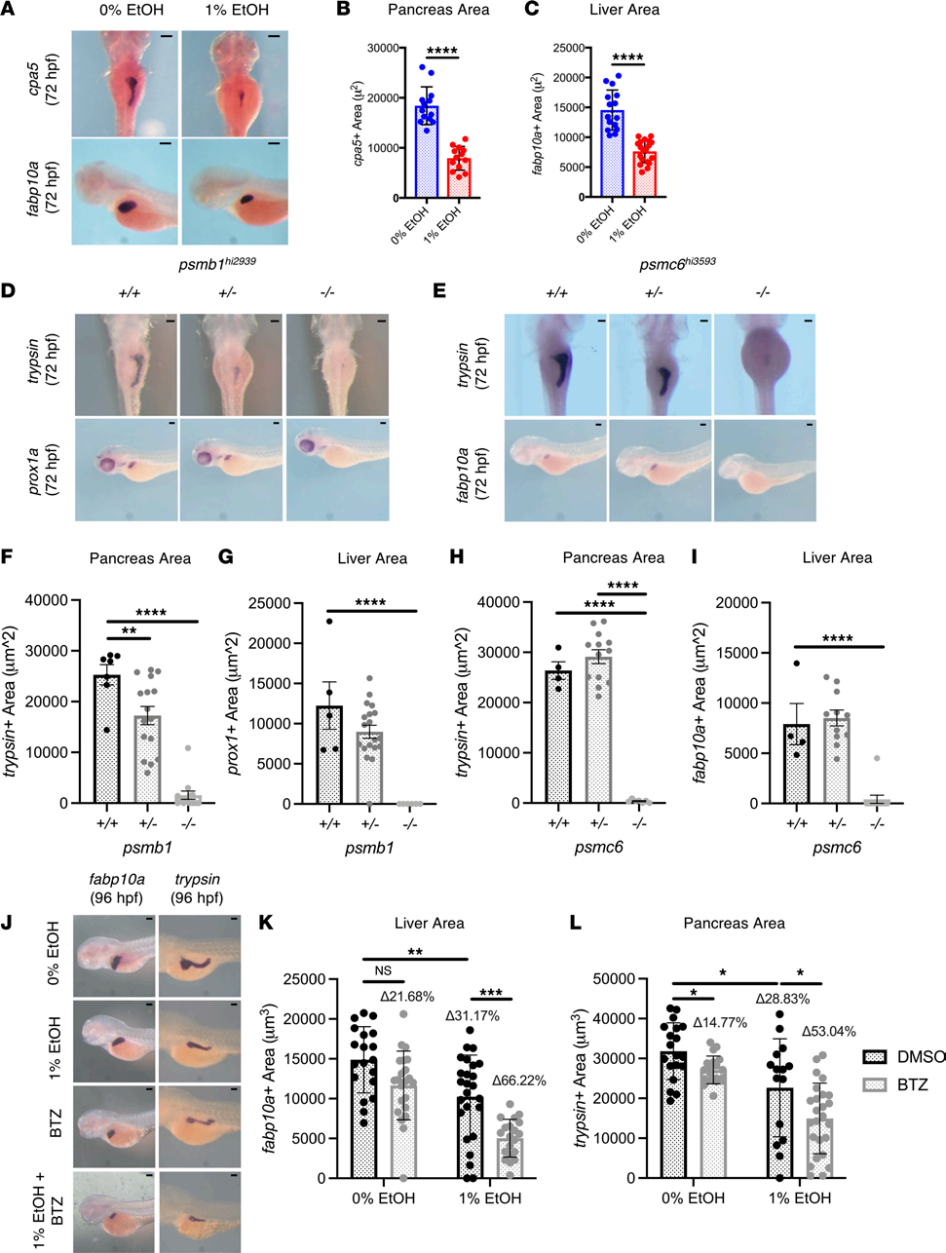
*psmb1* and *psmc6* are necessary for hepatopancreatic development. (**A**) ISH for exocrine pancreas marker *cpa5* and hepatocyte marker *fabp10a*. EtOH (1%) (12–72 hpf) reduced exocrine pancreas and liver size. (**B** and **C**) ImageJ quantification of pancreas and liver size (****P ≤ 0.0001, unpaired 2-tailed *t* test, *n* ≥13). (**D** and **E**) ISH for exocrine pancreas marker *trypsin* and hepatocyte markers *prox1a* and *fabp10a* in *psmb1^hi2939^* and *psmc6^hi3593^* (72 hpf) mutants. (**F**–**I**) Quantification of pancreas and liver size using ImageJ. *psmb1^+/–^*, *psmb1^–/–^*, and *psmc6^–/–^* have significantly reduced exocrine pancreas size. *psmb1^–/–^* and *psmc6^–/–^* have significantly reduced liver size (*n* > 4, ****P ≤ 0.0001, **P < 0.01, ordinary 1-way ANOVA with Dunnett’s multiple-comparison test). (**J**–**L**) ISH for liver marker *fabp10a* and exocrine pancreas marker *trypsin*, followed by quantification of organ size using ImageJ area calculation at 96 hpf. EtOH (1%) (12–96 hpf) significantly reduced liver and pancreas size. Treatment with BTZ significantly exacerbated the effects of EAE on liver and pancreas area (*P < 0.05, **P < 0.01, ***P < 0.001, 2-way ANOVA with Sidak’s multiple comparisons). BTZ concentration = 2.5 μM (12–96 hpf). Scale bars: 100 μm. From left, column sample *n* = 7, 16, 13 (**F**); *n* = 6, 20, 7 (**G**); *n* = 6, 20, 7 (**H**); *n* = 5, 12, 12 (**I**); *n* = 19, 21, 25, 19 (**K**); and *n* = 19, 19, 15, 25 (**L**). Data represent mean ± SD.

Pancreatic acinar cells and hepatocytes exhibit high levels of protein production relative to other cell types; therefore, both cell types require carefully balanced proteostasis (66, 67). We next sought to determine if liver and pancreas development is significantly altered by EAE via UPS function. Following exposure to EtOH during hepatopancreatic formation and differentiation (12–72 hpf) and metabolite MeCHO during hepatopancreas differentiation (56–78 hpf), larvae exhibited significantly reduced exocrine pancreas and liver size (Figure 7, A–C, and Supplemental Figure 6, A–C) (43). These findings demonstrate that EtOH and MeCHO impair liver and pancreas development. To determine whether proteotoxic stress can also detrimentally impact liver and pancreas development, we examined endoderm formation in *psmb1^hi2939^* and *psmc6^hi3593^* mutants. By ISH, *psmb1^–/–^* and *psmc6^–/–^* larvae had significant reductions in exocrine pancreas (*trypsin*) and liver (*prox1a*, *fabp10a*) size, with the liver and pancreas almost completely absent at 72 hpf (Figure 7, D–I). However, *psmb1^+/–^* showed no additional sensitivity to BTZ-induced pancreas size reduction compared with their WT siblings (Supplemental Figure 6, D and E). Interestingly, all *psmb1^hi2939^* genotypes exhibited normal hepatopancreatic progenitor development as marked by *hhex* and *foxa3* at 48 hpf, consistent with the observations in EAE larvae (Supplemental Figure 6F) (43). These data reveal that proteasome function is necessary for liver and pancreas maturation downstream of endoderm specification and indicate that *psmb1* may be specifically required for acinar cell maturation.

Using ISH for *fabp10a* and *trypsin*, we evaluated the impact of chemical proteasome inhibition on liver and pancreas size. Like treatment with 1% EtOH (12–96 hpf), BTZ exposure resulted in mild reductions in liver and pancreas size (Figure 7, J–L). Cotreatment with EtOH and BTZ resulted in more significant reductions in organ size than either exposure alone (Figure 7, J–L). Furthermore, the net reductions in liver and pancreas size in the combined EtOH plus BTZ treatment group were approximately 10% greater than the sum of net size reductions for EtOH or BTZ alone, indicating a synergistic effect (Figure 7, K and L). These findings reveal that the liver and pancreas are susceptible to EAE and that proteasome inhibition further exacerbates EAE-induced reductions in endodermal organ development. Individuals with genetic or environmental perturbations in the UPS may, therefore, be subject to increased endodermal-derived organ alterations caused by EAE.

## Discussion

Mechanisms leading to the development of FASD-related phenotypes are either unknown or poorly characterized. Here, we identify the UPS as a mechanistic target of EAE. EtOH disrupted protein homeostasis by altering proteasome subunit expression, increasing ubiquitylated protein load, and inhibiting chymotrypsin-like proteasome peptidase activity. Importantly, proteasome components were upregulated 48 hours after cessation of EtOH exposure, likely as a compensatory mechanism to manage increased demand, suggesting that impacts on protein homeostasis are persistent. To determine the impact of proteasome dysfunction, we characterized development in *psmb1^hi2939Tg^* and *psmc6^hi3593Tg^* mutants. Genetic loss of *psmb1* and *psmc6* produced numerous tissue-specific developmental defects, including craniofacial, endoderm, and nervous system abnormalities, resembling those that result from EAE. Furthermore, proteasome inhibition potentiated the effects of EAE and caused a higher prevalence of severe defects. Our work demonstrates that disruptions in the UPS serve as a cellular mechanism underlying organ malformations in FASDs.

An important observation of this study is that EAE leads to the abnormal expression of 20S, 19S, and 11S proteasome components in zebrafish. These data point to a role for the proteasome in the development of FASDs. While these observations were made in zebrafish, abnormal expression of proteasome components following PAE may be conserved in mammals. In previous studies, protein levels of PSMB7, PSMA6, and ubiquitin conjugating enzyme E2 N (UBE2N) were significantly downregulated in the murine fetal brain following EtOH exposure (68, 69). Microarray transcript profiling of the embryonic headfold 3 hours following maternal EtOH exposure in mice similarly resulted in a transcriptional downregulation of genes mapping to the proteasome (70). Following birth, the isolated cerebral cortex of weaning rats with PAE exhibited an upregulation of proteasome components PSMD11 and PSMA4 and a downregulation of proteins responsible for ubiquitylation (UBE2N) (71). Taken together, these studies collectively demonstrate that the UPS is perturbed by EtOH in utero, with alterations in protein expression and function maintained following birth. Further characterization of the spatiotemporal regulation of proteasome components during mammalian development, including in the context of PAE, could reveal novel roles for these factors in organogenesis.

An important conclusion from our study is that the impact of EtOH on the UPS may be generalizable regardless of age, such that much of what we have learned about adult cells could be instructive in understanding how EtOH impacts the developing fetus. In the adult mouse cerebral cortex, chronic EtOH consumption results in a significant increase in ubiquitylated protein and leads to the induction of the 20S immunoproteasome subunits and PA28 through proinflammatory cytokines and innate immune receptors in glial cells (72). Furthermore, in alcoholic liver disease, patients develop cytoplasmic inclusions consisting of ubiquitinated, aggregated protein known as Mallory bodies that are thought to result from failed proteasomal degradation of oxidized or otherwise damaged protein (48, 73, 74). Examining conserved mechanisms of impaired proteostasis in EtOH-exposed fetal and adult tissues could lead to the identification of interventions that could benefit patients with FASDs and alcohol use disorders.

In this study, we demonstrate that EtOH and MeCHO impair chymotrypsin-like proteasome peptidase activity during zebrafish development and that EtOH impairs chymotrypsin-like proteasome peptidase activity in murine hepatic organoids, but not BEC organoids. Prior studies have proposed that chronic, but not acute, treatment with EtOH impairs chymotrypsin-like proteasome activity in the adult liver, leading to proteotoxic stress and cellular dysfunction (50, 75). MeCHO-adducted cytosolic proteins can also impair proteasome function in vitro, as have MeCHO adducts on purified proteasomes (50). Our study suggests that even short-term exposures of EtOH and MeCHO can disrupt proteasome function, potentially in a multitude of cell types throughout development and even adulthood. Based on our data, we conclude that the ability to metabolize EtOH to MeCHO during development may render specific cell types, especially the liver and pancreas, susceptible to EtOH-induced injury. The mechanism whereby EtOH inhibits proteasome function during adulthood is unresolved; however, evidence points to the modification of proteasome components and their substrates with primary and secondary products derived from the oxidative metabolism of EtOH (76). In-depth biochemical analysis on purified 20S proteasomes would clarify the mechanisms by which EtOH and MeCHO can inhibit proteasome activity.

Studies have described neurodevelopmental disorder and birth defects resulting from genetic perturbation of the UPS in humans (77–79). However, the developmental implications of genetic proteasome perturbation have been poorly characterized in animal models because of early lethality. Our study takes advantage of external zebrafish development to examine organ development in *psmb1* and *psmc6* mutants in detail. An important observation from the characterization of these mutants is that specific cell types are susceptible to select proteasome component KO, while others are not. For example, neurons undergo dramatic apoptosis, whereas other cell types are preserved. The basis for this susceptibility may be relevant for understanding tissue-specific effects of EtOH. First, the proteasome is an adaptable complex made of many component proteins, and tissue-specific versions of the 20S proteasome have been discovered (80, 81). Inhibition of the proteasome may, therefore, disproportionately affect cell types that rely more heavily on specific proteasome components. Second, there is evidence that proteasome subunits may have nonproteolytic roles, such as in transcription initiation, chromatin remodeling, and transcription factor activity (82–84). Our study demonstrates that EtOH affects the expression of many components of the UPS. Theoretically, there may be nonproteolytic consequences of this expression change that are cell type specific, and this could be examined in future studies. Finally, the regulatory proteins whose expression and activity level are controlled by the UPS are involved in a number of fundamental cellular processes, including cell cycle progression, neuronal morphogenesis and synapse development, cell stress response, and cell differentiation (24, 85, 86). Failure to degrade these short-lived regulatory proteins in a highly regulated manner could disrupt critical cellular events in specific cell types but not others.

In summary, our study provides evidence that EtOH and its primary metabolite MeCHO disrupt protein homeostasis during development and that abnormal UPS function can contribute to the development of FASDs. Identifying the UPS as a critical target of EtOH during development may explain tissue-specific effects of EtOH during development and provide avenues for combating EtOH-induced tissue injury.

## Methods

### Animal studies.

Lines used in this study include WT (AB strain), gut:GFP, *Tg(fabp10a:dsRed; elastase:GFP)*, *psmb1^hi2939Tg^*, and *psmc6^hi3593Tg^* (56, 87–89). *psmb1^hi2939Tg^* and *psmc6^hi3593Tg^* zebrafish lines were generated by retrovirus-mediated insertional mutagenesis in Nancy Hopkins’ laboratory (MIT) and validated by PCR upon retrieval from ZIRC (56–59). For all embryonic and juvenile experiments, clutch-matched fish were randomly assigned to each treatment group and used without sex bias. For adult EtOH exposures, qPCR was performed on male livers. Each independent experiment was conducted in clutch-matched siblings; however, results were confirmed and validated in at least 2 clutches by 2 different authors.

### Chemical exposure.

Zebrafish larvae were exposed to 0%–1.0% EtOH or to 0% or 0.01% MeCHO dissolved in fish water during periods ranging from 12 hpf to 5 dpf. For longitudinal studies, fish were removed from EtOH at 5 dpf and transferred to system water. Pharmacological inhibition of the proteasome was accomplished by exposing embryos to 0–5.0 μM BTZ or 0–10 μM MG132 in system water. Livers from Tm-treated (0.5 μg/mL; Sigma-Aldrich, T7765) animals were dissected from euthanized fish and harvested for RNA at the completion of the 24-hour treatment. Adult zebrafish (1–1.5 years old) were exposed to 0% or 0.9% EtOH for 24 hours.

### RNA isolation for RNA-Seq.

For RNA-Seq, RNA was extracted in Invitrogen TRIzol (catalog 15596-026) from pooled (multiple combined clutches) whole 7 dpf AB larvae and purified using the Qiagen RNeasy Mini Kit (catalog 74104). RNA quality was verified on the Aligent Bioanalyzer, and DNA contamination was removed with the Thermo Fisher Scientific TURBO DNA-free kit (catalog AM1907). Single-end NextSeq Series High-Output RNA-Seq was performed on poly-A selected coding mRNAs at the Dana-Farber Center for Cancer Computational Biology.

### RNA-Seq and GSEA.

Larval RNA-Seq (7 dpf) reads were aligned to the GRCz10 reference assembly with the STAR aligner, and differential gene expression was performed with DESeq2 software using a negative binomial with a Wald test. GSEA was performed using the GOrilla GO enrichment analysis and visualization tool and the DAVID Bioinformatics Resources 6.8 Analysis Wizard (90–92). Heatmap visualization and hierarchical clustering was performed using the R package pheatmap with the default parameters.

### FACS.

*Tg(fabp10a:dsRed; elastase:GFP)* embryos were raised in 0% or 1% EtOH from 10 hpf to 120 hpf. Forty larvae were pooled per sample and dissociated with 2.5 μg/mL Liberase (Sigma-Aldrich, 05401119001) in a 600 rpm shaker for 30 minutes at 37°C. Samples were pipetted 10–15 times every 10 minutes and passed through a 40 μm filter to encourage a single-cell suspension. DAPI staining differentiated live from dead cells, and 8,000 viable dsRed^+^ hepatocytes were sorted by FACS per sample using a BD FACSAria Fusion Flow Cytometer (BD Biosciences) into buffer RLT with 1% β-mercaptoethanol. RNA was then isolated using the Qiagen RNeasy Micro Kit (Qiagen, 74004) and utilized for cDNA library construction and downstream qPCR.

### WB.

Proteins were extracted from pooled 72 hpf, 96 hpf, and 120 hpf AB larvae (30–35/replicate), quantified with the Pierce BCA Protein Assay Kit (Thermo Fisher Scientific) using the Molecular Devices SpectraMax M5 plate reader, and resolved using SDS-PAGE. For mutant analysis, 96 hpf homozygous *psmb1^hi2939Tg^* and *psmc6^hi3593Tg^* larvae were pooled and compared with a mixed pool of WT and heterozygote clutch-matched siblings. Antibodies included mouse anti-ubiquitin (P4D1) (1:1,000, Cell Signaling Technology, 3936), mouse anti–proteasome 20S α1, 2, 3, 5, 6, and 7 subunits (1:1,000, Enzo, BML-PW8195, a mix of antibodies against all listed subunits), mouse monoclonal anti–α-tubulin (1:500, Sigma-Aldrich, T9026), rabbit polyclonal anti-GRP78 BiP (1:1,000, Abcam, 21685), anti–rabbit IgG HRP–linked antibody (1:3,000, Cell Signaling Technology, 7074), and anti–mouse IgG HRP–linked antibody (1:3,000, Cell Signaling Technology, 7076). Following transfer but prior to primary antibody incubation, membranes were incubated in Ponceau S Stain solution for 15 minutes at room temperature, rinsed 3 times with distilled water, and photographed using a scanner. For ubiquitin level measurement and normalization, each lane in the 8-bit inverted images was evaluated for integrated density using the Region of Interest tool in ImageJ and normalized to corresponding lanes stained with Ponceau S to account for differences in protein loading.

### ISH.

ISH was conducted on 4% paraformaldehyde-fixed (PFA-fixed) embryos according to standard protocols using established and newly cloned probes (93). Endoderm development was examined using probes for *foxa3* (pan endoderm), *trypsin* (acinar cells), *prox1a* (hepatoblasts and hepatocytes), and *fabp10a* (hepatocytes) (94, 95). Liver and pancreas sizes were determined using the area calculation function in ImageJ. For ISH requiring novel probes, primers were designed from established cDNA sequences using the IDT PrimerQuest Tool (Supplemental Table 6). Following PCR amplification of relevant cDNA, probes were synthesized in a reaction with the Roche T7 RNA Polymerase and transcription buffer (catalog 10881767001), Sigma-Aldrich DIG RNA Labeling Mix (catalog 11277073910), and Promega RNasin Ribonuclease Inhibitors (catalog N2111). RNA probes were purified with the Zymo Research RNA Clean & Concentrator-5 (R1013) and resuspended in hybridization buffer.

### qPCR.

cDNA libraries were synthesized from Trizol/Chloroform isolated RNA using the Bio-Rad iScript cDNA Synthesis Kit (catalog 1708891). Reverse transcription PCR (RT-PCR) reactions were performed using the iScript RT Supermix for RT-PCR (catalog 1708841) with exon-exon junction spanning primers designed using NCBI Primer-BLAST (Supplemental Table 7). Primers were validated using efficiency calculation reactions. Relative expression levels for each experiment were calculated using the ΔΔCt method. Expression was normalized to *ef1α* or *tbp* as indicated. Each biological replicate for the larval experiments (whole animal and heads) represents a separate pool of approximately 35 embryos.

### Organoid cultures.

Primary hepatocytes and BECs were isolated from C57BL/6 mice and maintained in 3D organoid culture as previously described (96). Isolated cells were mixed with Geltrex and allowed to polymerize at 37°C for 30 minutes. Cells were cultured in a humidified 5% CO_2_ atmosphere at 37°C using conditioned media for organoid culture. BEC organoid media consisted of a 1:1 mixture of L-WRN conditioned media and fresh 2× Media. L-WRN conditioned media was generated as previously described and contained WNT3A, R-Spondin, and Noggin growth factors (96). 2× Media contained advanced DMEM/F12 medium (Invitrogen), 103 U/mL;103 μg/mL penicillin/streptomycin (Invitrogen), 2 mM L-glutamine, 2x N2-supplement (Invitrogen), 2Å B27 without Vitamin A supplement (Invitrogen), 20 mM nicotinamide (Sigma-Aldrich), 0.002 mM dexamethasone (Sigma-Aldrich), 10 mM HEPES (Invitrogen), 1.25mM N-acetylcysteine (MilliporeSigma), 10 nM gastrin (MilliporeSigma), 20 μM Y27632 (Sigma-Aldrich, only upon initial derivation and upon passage), 50 ng/mL rmEGF (R&D Systems), 40 ng/mL rmHGF (Peprotech), and 1:500 Primocin (Invivogen). Hepatocyte media was the same as above, supplemented with 3 mM CHIR99021 (MilliporeSigma), 50 ng/mL FGF7 (Peprotech), and 50 ng/mL FGF10 (Peprotech) (97).

### Proteasome activity assays.

Hepatic and biliary organoid cultures were plated in 2D at equal densities (3,000 cells/well) on Corning 96-well, flat, clear-bottom, black, polystyrene TC-treated microplates (catalog 3603) that were coated in matrigel (200 μL of matrigel in 12.5 mL of cold DMEM/F12 for 30 minutes in 37°C incubator). Cells were treated with 0–100 mM EtOH overnight with and without 5 nM BTZ in 5 biological replicates per condition. Proteasome activity was measured using the Sigma-Aldrich Proteasome 20S Activity Assay Kit (MAK172), and relative fluorescence intensity was detected using the Molecular Devices SpectraMax M5 plate reader (λ_ex_, 490 nm; λ_em_, 525 nm) for 8 hours at 37°C. Experimental samples were blanked to a well containing coating and media. To account for fluorescence signal not generated by the 26S proteasome, signal from wells treated with 0 and 100 mM EtOH were normalized to matched wells treated with 5 nM BTZ. Zebrafish proteasome activity assays were performed on BCA assay-normalized cell extracts (~100 μg protein) as previously described (98). Approximately 35 whole larvae were homogenized per replicate in lysis buffer (50 mM HEPES [pH 7.8], 10 mM NaCl, 1.5 mM MgCl_2_, 1 mM EDTA, 1 mM EGTA, 250 mM sucrose, 5 mM DTT), and 5 biological replicates were included per group. Assay buffer (50 mM HEPES [pH 7.8], 10 mM NaCl, 1.5 mM MgCl_2_, 1 mM EDTA, 1 mM EGTA, 250 mM sucrose, 5 mM DTT, 2 mM ATP) contained 100 μM LLVY-AMC or Z-LLE–AMC substrate. Fluorescence intensity was monitored using the Molecular Devices SpectraMax M5 plate reader (λ_ex_, 360 nm; λ_em_, 460 nm) for 60 minutes at 37°C.

### Genotyping reactions.

DNA was isolated from *psmb1^hi2939Tg^* and *psmc6^hi3593Tg^* adult tail clips and PFA-fixed embryos via a NaOH/Tris extraction. For the NaOH/Tris DNA extraction, individual embryos or tail clips were incubated in a PCR plate with 50 mM NaOH (with 50 μL for embryos, 100 μL for tail clips) at 95°C for 30 minutes and cooled to 4°C. One-tenth volume (5–10 μL) of 1M Tris (pH 8) was added to each well, and the samples were centrifuged at 1,342*g* at room temperature for 15 minutes. To identify whether each fish carried a transgenic insertion, 2 PCR primers were utilized — one matching the native gene sequence and the other matching the insertion sequence. To detect the transgenic insertion, a *psmb1* forward (F) primer (5′-ATGATTTCTGCCCAGGCTTAT-3′) or *psmc6* reverse (R) primer (5′-TTCAGCACTTCACCGACAAT-3′) was utilized, along with a *lacZ* construct primer (5′-GGACGCGCGAATTGAATTATG-3′). To detect the WT allele, the *psmb1* F primer and *psmc6* R primer were utilized, along with a corresponding primers *psmb1* R (5′-GGATGCTGTAACCTTCACTTAAAC-3′) and *psmc6* F (5′-TGAGAGAGCAGCTGAAGGA-3′).

### Alcian blue stain.

Craniofacial cartilage was detected in 4 dpf zebrafish larvae using an adapted acid-free Alcian blue staining protocol (99). Briefly, larvae were fixed overnight in 4% PFA, washed in PBT, incubated in 50% EtOH for 10 minutes, and stained overnight in Alcian blue staining solution. Larvae were then washed in H_2_O, bleached (KOH, H_2_O, Tween-20, and H_2_O_2_), fixed in 4% PFA, and stored in glycerol for visualization. Images were taken with a Zeiss Discovery V8 stereoscope, and cartilage lengths were measured using ImageJ.

### IHC.

Both *psmb1^hi2939Tg^* and *psmc6^hi3593Tg^* embryos were fixed in 4% PFA for 2 hours at 21°C, stepped into 100% methanol, and stored overnight at –20°C. Embryos were then rehydrated into 1× phosphate-buffered saline with Tween (PBT), bleached, permeabilized with 20 μg/mL proteinase K for 10 minutes, and fixed in 4% PFA for 20 minutes. Next, embryos were repermeabilized with 0.5% Triton-X 100 for 2 hours, blocked (5% normal goat serum [NGS], 5% BSA, in PBT) for 1 hour, and incubated overnight with primary antibodies at 4°C. Embryos were then washed in PBT and incubated overnight in secondary antibodies at 4°C prior to imaging. Antibodies — including mouse anti-acetylated tubulin (Sigma-Aldrich, T7451), mouse anti-HuC/HuD (Invitrogen, A-21271), and goat anti–mouse IgG (H+L) cross-adsorbed secondary antibody Alexa Fluor Plus 488 (Thermo Fisher Scientific, A32723) — were used at a dilution of 1:250. Embryos were imaged with a Zeiss LSM 880 confocal microscope using a 10×/0.3 NA EC Plan-Neofluar objective lens.

### AO and TUNEL staining.

The 100× AO stock (1 mg/mL) was created in sterile water. Live embryos were stained in 1× AO solution in E3 embryo media for 30 minutes in the dark, washed 3× in E3 embryo media, and mounted in agarose for live confocal imaging. TUNEL staining was completed on 4% PFA fixed embryos using the TMR Red In Situ Cell Detection Kit (Roche, 12156792910). Embryos were mounted in agarose and imaged with a Zeiss LSM 880 confocal microscope using a 10×/0.3 NA EC Plan-Neofluar objective lens. Fluorescent punctae were counted using ImageJ.

### Data availability.

The data discussed in this publication have been deposited in NCBI’s Gene Expression Omnibus (GEO; ref. 100) and are accessible through GEO accession no. GSE172111.

### Statistics.

To determine whether there is a statistically significant difference between samples with only 1 independent variable, such as EtOH or genotype, 1-way ANOVA, unpaired 2-tailed Student’s *t* test, or unpaired 1-tailed Student’s *t* test was utilized. One-way ANOVA with Dunnett’s multiple-comparison test was used to confirm significant differences across 3 or more samples. For multiple comparisons spanning treatment conditions on 2 × 2 experimental designs where 2 independent variables may impact the dependent variable, 2-way ANOVA with multiple comparisons was utilized. Two-way ANOVA was also used to determine whether there was a significant interaction between independent variables EtOH and proteasome inhibitor treatment. For assessing differences in rate of outcome, such as percent survival or edema prevalence, 2-sided Fischer’s exact test was used. For all experiments, *P* > 0.05 was considered significant.

### Study approval.

All animal studies were approved by the IACUC at the Beth Israel Deaconess Medical Center (IACUC-BIDMC 056-2015) and the Brigham and Women’s Hospital (2016N000405).

## Author contributions

Conceptualization was contributed by OW and WG. Methodology was contributed by OW and WG. Validation of results was contributed by OW, BMM, and BJPM. Formal analysis was contributed by OW, BMM, BJPM, and IMO. Investigation was contributed by OW, BMM, BJPM, SHF, and CJS. Writing of the original draft was contributed by OW, WG, and TEN. Visualization was contributed by OW, SHF, and BMM. Supervision was contributed by WG. Project administration was contributed by OW and WG. Funding acquisition was contributed by WG and OW.

## Supplementary Material

Supplemental data

Supplemental table 1

Supplemental table 2

Supplemental table 3

## Version 1 12/08/2022

Electronic publication
