# Supplementary material for: Embryonic alcohol exposure disrupts the ubiquitin-proteasome system
Source: JCI Insight. 2022 Dec 8;7(23):e156914. doi: 10.1172/jci.insight.156914 (PMC9746913; doi:10.1172/jci.insight.156914)
Supplement: Supplemental data [file jciinsight-7-156914-s040.pdf]

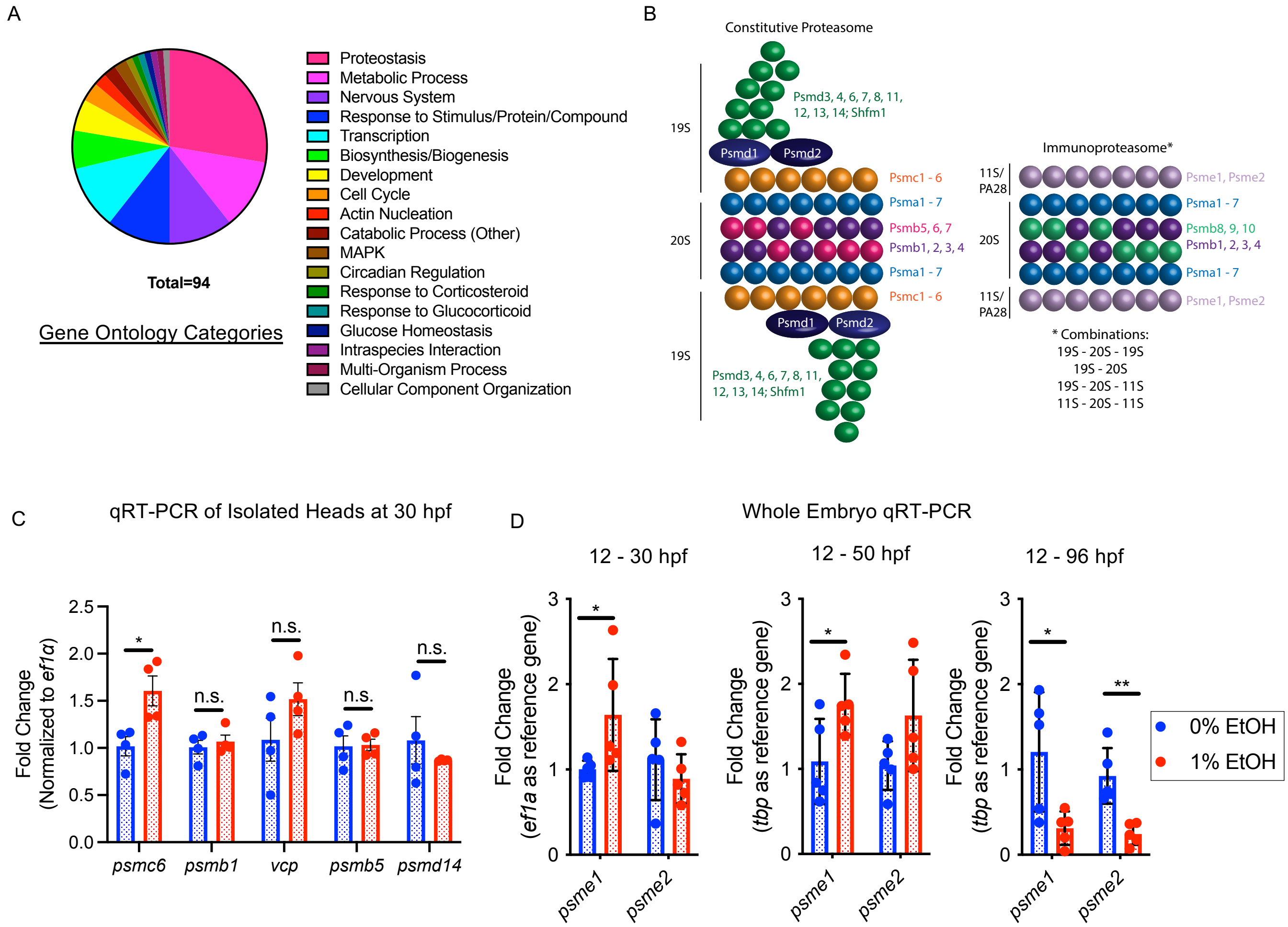

Figure S1. Related to Figure 1. RNA sequencing identifies the ubiquitin proteasome system as a target of embryonic alcohol exposure. A. Gene ontology categories from GSEA performed on RNA sequencing from whole 7 dpf 0% and 1% EtOH (12 hpf – 5 dpf) treated larvae. Go-terms within the category of "proteostasis" occur the most frequently, along with those relevant to "metabolic processes" and the nervous system. **B.** Schematic of the constitutive proteasome and the immunoproteasome components, adapted from McCarthy and Weinberg, 2015, Díaz-Villanueva et al., 2015, Murata et al., 2009, and Skerget et al., 2013. **C – D.** qRT-PCR analysis of surgically isolated heads (C) and whole embryo extracts (D) for expression of proteasome components at 30, 50 and 96 hpf (*$p < 0.05$, **$p \leq 0.01$, unpaired two-tailed t-test). Expression was normalized to *tbp* or *ef1α* as indicated. $n \geq 4$ per condition. Data represent mean ± SEM.

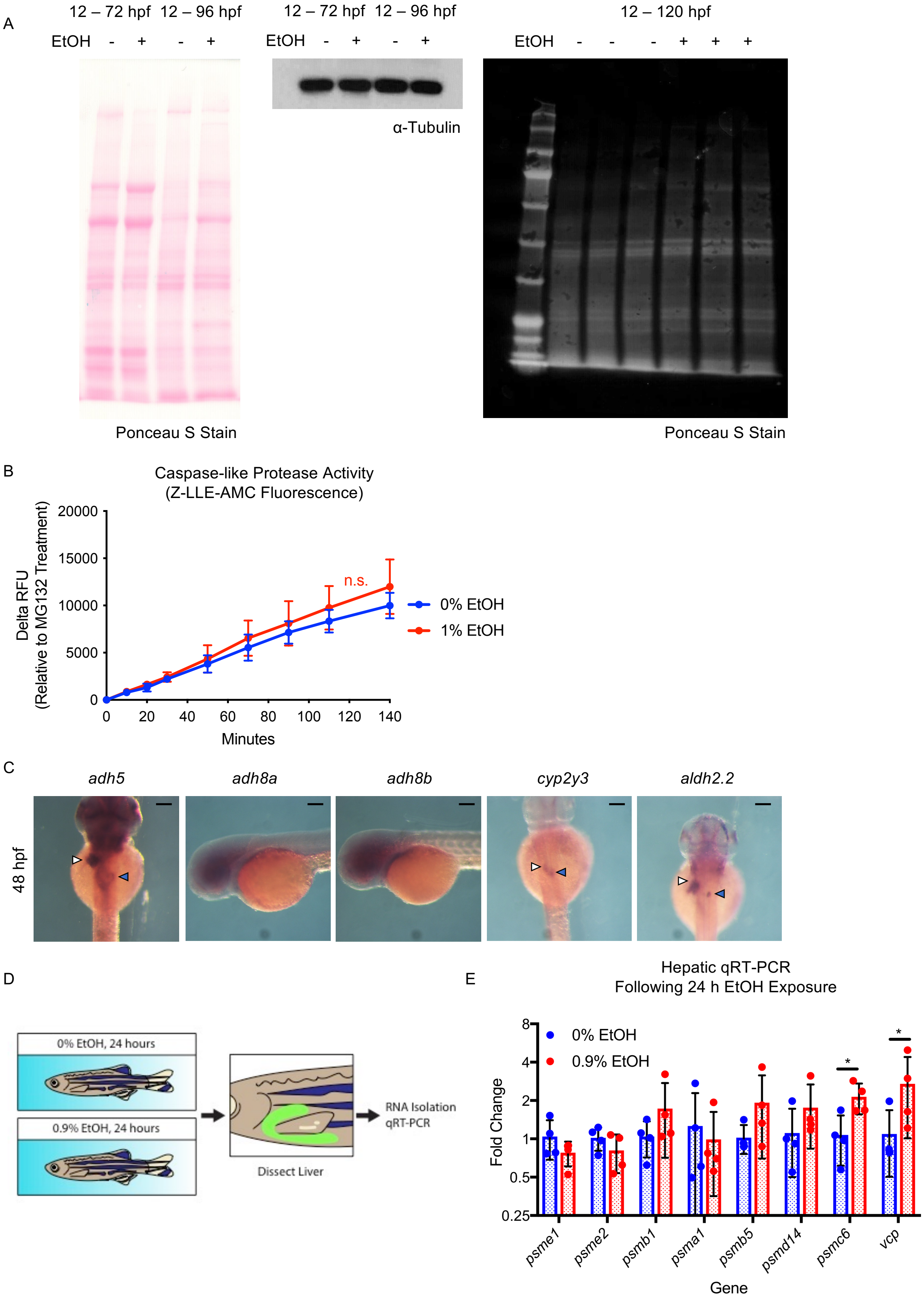

A
12 – 72 hpf 12 – 96 hpf
EtOH - + - +
Ponceau S Stain
12 – 72 hpf 12 – 96 hpf
EtOH - + - +
α-Tubulin
12 – 120 hpf
EtOH - - - + + +
Ponceau S Stain
B
Caspase-like Protease Activity
(Z-LLE-AMC Fluorescence)
Delta RFU
(Relative to MG132 Treatment)
20000
15000
10000
5000
0
n.s.
0% EtOH
1% EtOH
0 20 40 60 80 100 120 140
Minutes
C
adh5
adh8a
adh8b
cyp2y3
aldh2.2
48 hpf
D
0% EtOH, 24 hours
0.9% EtOH, 24 hours
Dissect Liver
RNA Isolation
qRT-PCR
E
Hepatic qRT-PCR
Following 24 h EtOH Exposure
Fold Change
8
4
2
1
0.5
0.25
0% EtOH
0.9% EtOH
*
*
psme1
psme2
psmb1
psma1
psmb5
psmd14
psmc6
vcp
Gene

Figure S2.

Figure S2. Related to Figure 2. EtOH modulates the ubiquitin proteasome system and proteasome peptidase activity in a cell-type specific manner. A. Ponceau S and α-tubulin-stained loading control from western blot transfer following 0% or 1% EtOH exposure from 12 hpf to 48, 72, and 120 hpf. Loading controls correspond to blots from Figure 2A. **B.** Caspase-like proteasome peptidase activity in whole larval extract at 5 dpf is not impacted by 1% EtOH (12 hpf – 5 dpf; n = 5 per condition). Data represent mean with standard deviation. **C.** ISH for *adh5, adh8a, adh8b, cyp2y3,* and *aldh2.2* at 48 hpf. Expression is detected in the brain and hepatopancreatic progenitor cells (pancreatic progenitors = blue arrow; liver progenitors = white arrow). Scale bars = 100 μm. **D.** Schematic of adult zebrafish EtOH exposure and liver isolation. **E.** qRT-PCR on isolated hepatic tissue reveals that EtOH significantly upregulates *psmc6* and *vcp* ($p < 0.05$, two-sided t-test). n = 4 per condition. Data represent mean ± SEM. Expression was normalized to *ef1α.*

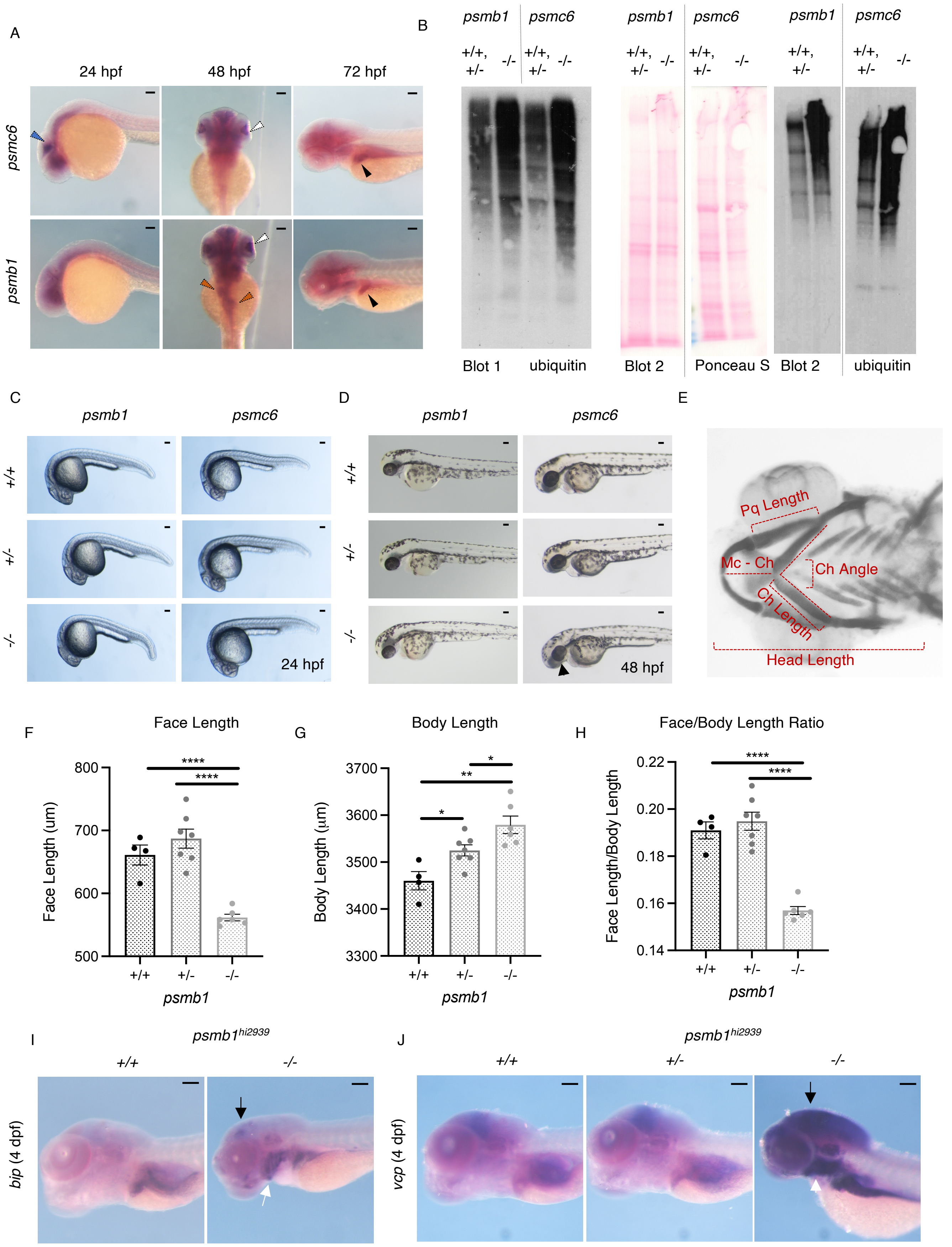
A
24 hpf
48 hpf
72 hpf
psmc6
psmb1
B
psmb1
psmc6
+/+, +/-
-/-
Blot 1
ubiquitin
Blot 2
Ponceau S
Blot 2
ubiquitin
C
psmb1
psmc6
+/+
+/-
-/-
24 hpf
D
48 hpf
E
Pq Length
Mc - Ch
Ch Angle
Ch Length
Head Length
F
Face Length
Face Length (um)
800
700
600
500
+/+
+/-
-/-
psmb1
G
Body Length
Body Length (μm)
3700
3600
3500
3400
3300
H
Face/Body Length Ratio
Face Length/Body Length
0.22
0.20
0.18
0.16
0.14
I
psmb1hi2939
bip (4 dpf)
J
vcp (4 dpf)

Figure S3.

Figure S3. Related to Figure 4. Psmb1 and Psmc6 are required for craniofacial and nervous system development. A. ISH for *psmc6* and *psmb1* reveals expression in the head, brain (blue arrow), eye (white arrows), hepatopancreatic progenitors (red arrows), and liver (black arrows). **B.** WB for ubiquitin in pooled 96 hpf *psmb1*$^{-/-}$ and *psmc6*$^{-/-}$ larvae relative to pooled wildtype and heterozygote siblings. Ponceau S stain included as a loading control. **C – D.** Brightfield imaging of *psmb1* and *psmc6* embryos at 24 hpf and 48 hpf. Homozygous mutants are not distinguishable from wildtypes and heterozygotes at 24 hpf. At 48 hpf, *psmc6* homozygotes can be distinguished by abnormal eye shape (black arrow). **E.** Map of craniofacial measurements performed on ventral-viewed 4 dpf larvae throughout the paper. **F – H.** Face length, body length, and face to body length ratio in *psmb1 +/+, +/-,* and *-/-* embryos at 4 dpf. *psmb1* homozygotes have reduced face length, increased body length, and a reduced face to body length ratio (unpaired two-tailed t-test,$^{*}p \leq 0.05$, $^{**}p \leq 0.01$, $^{****}p \leq 0.0001$). **I – J.** ISH for *vcp* and *bip* in *psmb1*^{hi2939} larvae at 4 dpf. Homozygous mutation increases *vcp* expression in the brain (black arrow), lower jaw (white arrow), and gut tube; similarly, *bip* expression is increased in the brain (black arrow) and lower jaw (white arrow). Scale bars = 100 µm. Data represent mean $\pm$ SEM. For F: from left to right, column sample numbers are n = 4, 7, 6. For G, n = 4, 7, 6. For H, n = 4, 7, 6.

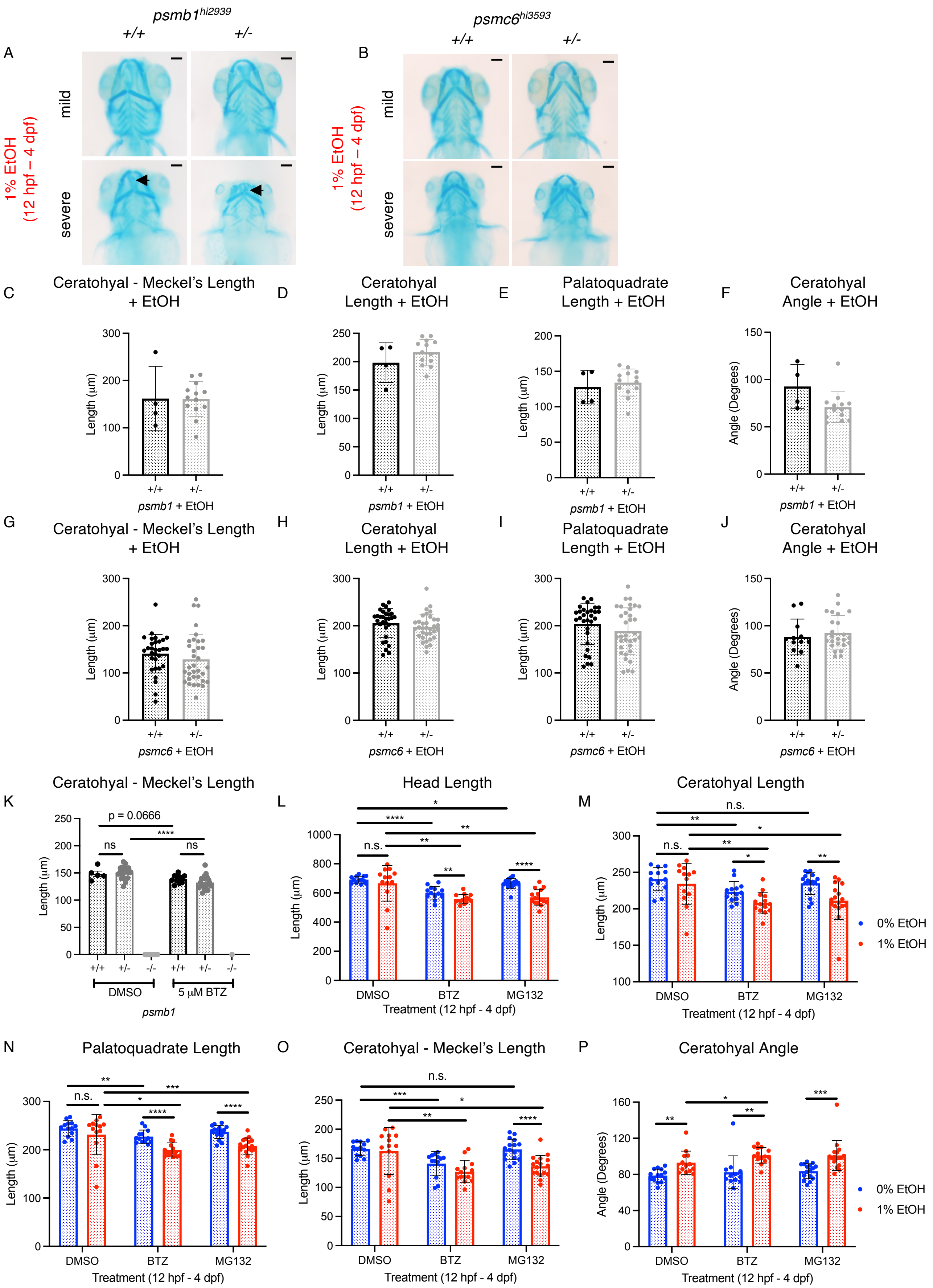

psmb1 hi2939
+/+
+/-
A
mild
severe
1% EtOH (12 hpf – 4 dpf)
psmc6 hi3593
B
C
Ceratohyal - Meckel's Length + EtOH
D
Ceratohyal Length + EtOH
E
Palatoquadrate Length + EtOH
F
Ceratohyal Angle + EtOH
Length (µm)
Angle (Degrees)
psmb1 + EtOH
G
H
I
J
psmc6 + EtOH
K
Ceratohyal - Meckel's Length
p = 0.0066
ns
****
-/-
DMSO
5 µM BTZ
psmb1
L
Head Length
M
Ceratohyal Length
n.s.
0% EtOH
1% EtOH
BTZ
MG132
Treatment (12 hpf - 4 dpf)
N
Palatoquadrate Length
O
Ceratohyal - Meckel's Length
P
Ceratohyal Angle

Figure S4.

Figure S4. Related to Figure 5. **Craniofacial structure in *psmb1*^{hi2939} and *psmc6*^{hi3593} mutants in response to EtOH exposure. A – B.** Alcian blue stain of cartilage in 4 dpf *psmb1*^{hi2939} and *psmc6*^{hi3593} larvae treated with 1% EtOH (12 hpf – 4 dpf). Wildtype and heterozygous siblings both develop EtOH-induced craniofacial abnormalities, such as facial shortening and Meckel's cartilage abnormalities (black arrows). Scale bars = 100 µm. **C – J.** Craniofacial measurements in 4 dpf *psmb1*^{hi2939} and *psmc6*^{hi3593} larvae after 1% EtOH exposure (12 hpf – 4 dpf). **K.** The distance between ceratohyal to Meckel's cartilage in *psmb1*$^{+/+}$ and *psmb1*$^{+/-}$ DMSO and BTZ treated embryos. No significant differences are observed between wildtypes and heterozygotes in the BTZ treatment group, suggesting equal sensitivity. **L – P.** Craniofacial measurements in 4 dpf Alcian blue stained larvae, including head length, ceratohyal length, palatoquadrate length, distance between ceratohyal to Meckel's cartilage, and ceratohyal angle. Measurements were calculated in ImageJ from a ventral view of the head skeleton. For K – P: *p < 0.05, **p $\leq$ 0.01, ***p $\leq$ 0.001, ****p $\leq$ 0.0001, multiple t-tests using Holm-Sidak. Data represent mean $\pm$ SEM. For C – F, n = 4 for *psmb1*$^{+/+}$ and n = 13 for *psmb1*$^{+/-}$. For G – J, n $\geq$ 12 per genotype. For L – P, n $\geq$ 13 per condition.

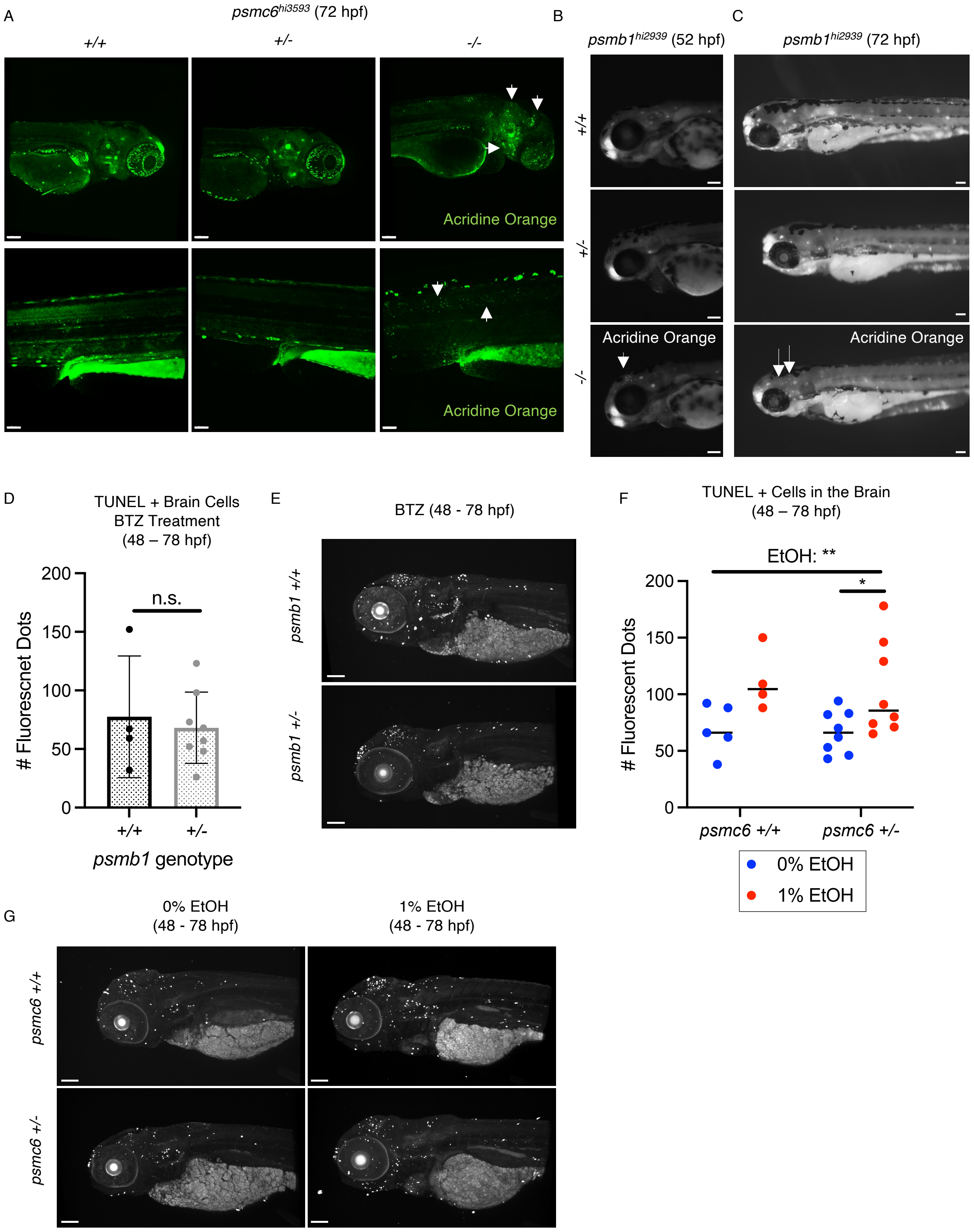

A
psmc6hi3593 (72 hpf)
+/+
+/-
-/-
Acridine Orange
Acridine Orange
B
psmb1hi2939 (52 hpf)
C
psmb1hi2939 (72 hpf)
+/+
+/-
-/-
Acridine Orange
Acridine Orange
D
TUNEL + Brain Cells
BTZ Treatment
(48 – 78 hpf)
n.s.
Fluorescnet Dots
200
150
100
50
0
+/+
+/-
psmb1 genotype
E
BTZ (48 - 78 hpf)
psmb1 +/+
psmb1 +/-
F
TUNEL + Cells in the Brain
(48 – 78 hpf)
EtOH: **
*
Fluorescent Dots
psmc6 +/+
psmc6 +/-
0% EtOH
1% EtOH
G
0% EtOH
(48 - 78 hpf)
1% EtOH
(48 - 78 hpf)
psmc6 +/+
psmc6 +/-

Figure S5.

Figure S5. Related to Figure 6. Psmb1 and Psmc6 are required for cell survival in the developing brain, spinal cord, and pharyngeal arches. A. Confocal image analysis of acridine orange (AO) stained *psmc6*^{hi3593} (72 hpf) mutants reveals increased apoptosis in brain and spinal cord (white arrows). Photographs are quantified in Figure 6B-C. **B – C.** Acridine orange stain in *psmb1*^{hi2939} larvae. Homozygotes have excess staining in the brain at 52 and 72 hpf (white arrows). **D – E.** TUNEL staining and quantification of 2.5 μM BTZ (28 – 78 hpf) treated *psmb1*$^{+/+}$ and *psmb1*$^{+/-}$ embryos. No significant difference in staining was observed, indicating that heterozygotes are not sensitized to neuronal death following BTZ treatment. For D, n = 4 (+/+) and n = 8 (+/-). **F – G.** TUNEL staining after exposure of *psmc6*$^{+/+}$ and *psmc6*$^{+/-}$ to 1% EtOH from 48 – 78 hpf. EtOH significantly increased the number of apoptotic cells in the brain; however, there was no significant effect of genotype on the amount of apoptosis observed (*$p < 0.05$, **$p \leq 0.01$, two-way ANOVA with Sidak's multiple comparisons test). For F, from left to right, n = 5, 4, 8, 8. Scale bars = 100 μm. Data represent mean ± SEM.

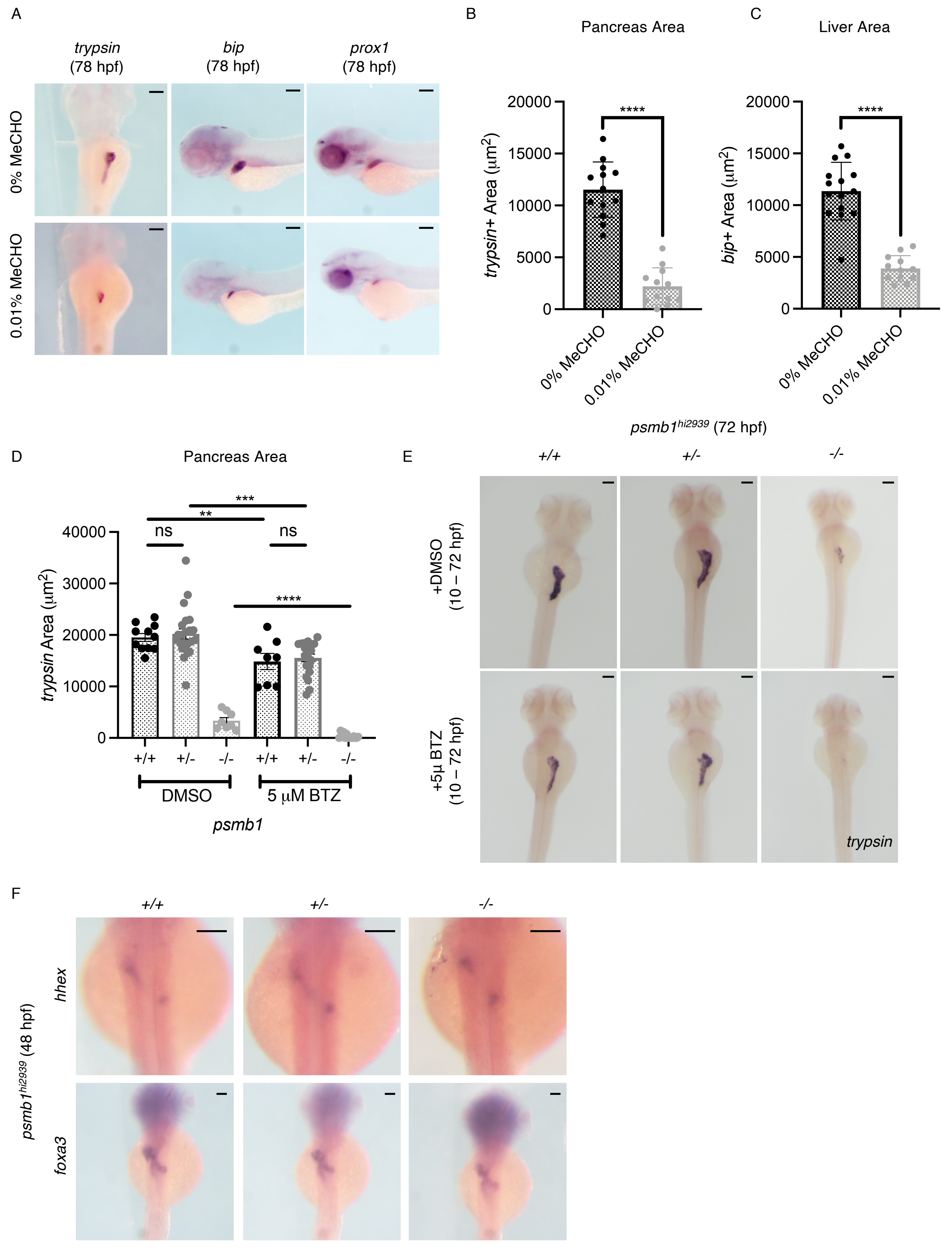
A
trypsin (78 hpf)
bip (78 hpf)
prox1 (78 hpf)
0% MeCHO
0.01% MeCHO
B
Pancreas Area
trypsin+ Area (µm2)
20000
15000
10000
5000
0
****
0% MeCHO
0.01% MeCHO
C
Liver Area
bip+ Area (µm2)
20000
15000
10000
5000
0
****
0% MeCHO
0.01% MeCHO
psmb1hi2939 (72 hpf)
D
Pancreas Area
trypsin Area (µm2)
40000
30000
20000
10000
0
ns
ns
**
***
****
+/+
+/-
-/-
DMSO
5 µM BTZ
psmb1
E
+/+
+/-
-/-
+DMSO (10 – 72 hpf)
+5µ BTZ (10 – 72 hpf)
trypsin
F
+/+
+/-
-/-
psmb1hi2939 (48 hpf)
hhex
foxa3

Figure S6.

Figure S6. Related to Figure 7. Hepatopancreatic development in MeCHO treated embryos and proteasome mutants. A. ISH for *trypsin* (pancreas), *bip,* and *prox1* (liver) in embryos treated with 0% or 0.01% MeCHO from 56 – 78 hpf. MeCHO reduced the size of the pancreas and liver without causing a dramatic upregulation in *bip* in additional tissues. **B – C.** Quantification of pancreas and liver size using Image J (****$p \leq 0.0001$, two-sided t-test). For B – C, from left to right, n = 19, 18, 20, 17. **D.** Pancreas area in *psmb1*$^{+/+}$ and *psmb1*$^{+/-}$ DMSO and BTZ treated embryos. No significant differences are observed between wildtypes and heterozygotes in the BTZ treatment group, suggesting equal sensitivity (**$p \leq 0.01$, ***$p \leq 0.001$, ****$p \leq 0.0001$ unpaired two-sided t-test, confirmed with Brown-Forsythe and Welch ANOVA tests). From left to right, n = 11, 23, 8, 8, 20, 14. **E.** ISH for *trypsin* (pancreas) in 72 hpf *psmb1*^{hi2939} embryos following DMSO and BTZ treatment. **F.** ISH for hepatoblast marker *hhex* and pan-endoderm marker *foxa3* in *psmb1* mutants. No notable differences are observed between genotypes. Scale bars = 100 μm. Data represent mean ± SEM.

Condition	Alive	Dead	% Survival	p-value*
0% EtOH	46	0	100	
1% EtOH	54	0	100	>0.9999
0% EtOH + 2.5 µM BTZ	54	0	100	>0.9999
1% EtOH + 2.5 µM BTZ	27	20	57.45	<0.0001

* Fischer's exact test (two-sided)

Table S4. Related to Figure 5A. Larval survival at 4 dpf after treatment with 1% EtOH (12 hpf – 4 dpf) or 2.5 µM BTZ.

Condition	normal	cardiac edema	global edema	% any edema	p-value*
0% EtOH DMSO	49	1	0	2	
0% EtOH 0.5 µM BTZ	54	0	0	0	0.4808
0% EtOH 1 µM BTZ	44	1	0	2	>0.9999
0% EtOH 2.5 µM BTZ	25	15	0	38	<0.0001
0.5% EtOH DMSO	48	2	2	8	0.3629
0.5% EtOH 0.5 µM BTZ	35	8	4	26	0.0007
0.5% EtOH 1 µM BTZ	30	10	2	29	0.0004
0.5% EtOH 2.5 µM BTZ	32	17	2	37	<0.0001
1% EtOH DMSO	26	6	2	24	0.0026
1% EtOH 0.5 µM BTZ	23	14	5	45	<0.0001
1% EtOH 1 µM BTZ	33	13	5	35	<0.0001
1% EtOH 2.5 µM BTZ	12	29	4	73	<0.0001

* Fischer's exact test (two-sided)

Table S5. Related to Figure 5B. Edema prevalence in 5 dpf larvae exposed to a EtOH, BTZ, or both in increasing concentrations.

Probe Primer	Sequence (5' - 3')
adh5 Forward	GAACACGCTCCTCTGGATAAA
adh5 Reverse	GAAATTAATACGACTCACTATAGGAGACAAAGGTGACCGTGATTAG
adh8a Forward	TTGTCACACCGACCTTTACC
adh8a Reverse	GAAATTAATACGACTCACTATAGGTCCAGCGCACTTCTCATTAC
adh8b Forward	CTGGCCGAGTACATCGTTATTAG
adh8b Reverse	GAAATTAATACGACTCACTATAGGCCACTCCTCCATTGGTCATTT
aldh2.1 Forward	GTGTGGAGAGAGCCAAGAATAG
aldh2.1 Reverse	GAAATTAATACGACTCACTATAGGCCTGAACTCCGAACACGTTATAG
aldh2.2 Forward	CCAACCATCAATCCTGCTACT
aldh2.2 Reverse	GAAATTAATACGACTCACTATAGGGAGCTCCAGTGTGACTTTCTT
crestin Forward	GCTGCCAAAGAGGAGATTGA
crestin Reverse	GAAATTAATACGACTCACTATAGGGAGGTGAAGAGGTGCTGTTTAG
cyp2y3 Forward	GGAGCAGTGGATTCAAGAAGAG
cyp2y3 Reverse	GAAATTAATACGACTCACTATAGGCAGAGTGCAGCATGGGAATAA
neuroD Forward	GGGTCCCAAGAAGAAGAAGATG
neuroD Reverse	GAAATTAATACGACTCACTATAGGCGTGTCGAGCAGTCTGATAAA
bip Forward	TTTGCCGAAGAGGACGATAAG
bip Reverse	GAAATTAATACGACTCACTATAGGCTTCATAGTGGAGCGGAACAA
vcp Forward	CCAGCTCTCTTCAAGGCTATT
vcp Reverse	GAAATTAATACGACTCACTATAGGGCCACCATCACCAACATTTC
psmb1 Forward	ATGATTTCTGCCCAGGCTTAT
psmb1 Reverse	GAAATTAATACGACTCACTATAGGCTCCACGTTCTCCATGTTCTT
psmc6 Forward	GTCGCAGACAGCTGGATAAA
psmc6 Reverse	GAAATTAATACGACTCACTATAGGGAAATCCTCCTGGGTCACATAC

Table S6. Related to Materials and Methods "in situ hybridization" section. Probe primer sequences.

qPCR Primer	Sequence (5' - 3')
tbp Forward	CGGTGGATCCTGCGAATTA
tbp Reverse	TGACAGGTTATGAAGCAAAACAACA
ef1a Forward	GCGTCATCAAGAGCGTTGAG
ef1a Reverse	TTGGAACGGTGTGATTGAGG
psme1 Forward	GATTCGCAACACTTACGCCA
psme1 Reverse	CATCAGCCTGGTTGCTCAGT
psme2 Forward	TAAACGTCTGCGTCTCTCTGC
psme2 Reverse	TGGCGGTAGTTTTCTATCCTCAC
psmb5 Forward	ACAAAAGAGGGCCAGGACTC
psmb5 Reverse	TCAAGTCGTATCGAAGGCCG
vcp Forward	ACGAGACCATTGACGCAGAG
vcp Reverse	TCTGGCTAAGAGCCCACCTA
psma1 Forward	GCGTCAGGAGTGTTTGGACT
psma1 Reverse	TGAGTCTTGCTGCCGATGAG
psma5 Forward	TCTCACATTGGCTGTGCCAT
psma5 Reverse	AACGGCTTGTGTTACGCTCT
psmb7 Forward	CTTGGGCTCAGGCAGTAACA
psmb7 Reverse	AACTTCCGGTTCTGACACCC
psmb1 Forward	TGTCCACCATTCTGTACGGC
psmb1 Reverse	TAAACAGCACCTCGACCCTC
psmc6 Forward	CAGAGTGTGGGGCAGATTGT
psmc6 Reverse	CCACTCTAGTGCCAGGCTTC
psmd14 Forward	AGCGTCAAGGGAAAGGTTGT
psmd14 Reverse	GGTTCATGACCCAGCACCAT
bip Forward	ATCAGATCTGGCCAAAATGC
bip Reverse	CCACGTATGACGGAGTGATG

Table S7. Related to Methods “qRT-PCR” section. qRT-PCR primer sequences.
